# NLRP3 Cys126 palmitoylation by ZDHHC7 promotes inflammasome activation

**DOI:** 10.1016/j.celrep.2024.114070

**Published:** 2024-04-06

**Authors:** Tao Yu, Dan Hou, Jiaqi Zhao, Xuan Lu, Wendy K. Greentree, Qian Zhao, Min Yang, Don-Gerard Conde, Maurine E. Linder, Hening Lin

**Affiliations:** 1 Howard Hughes Medical Institute, Department of Chemistry and Chemical Biology, Cornell University, Ithaca, NY 14853, USA; 2 Department of Chemistry and Chemical Biology, Cornell University, Ithaca, NY 14853, USA; 3 Department of Molecular Medicine, Cornell University, Ithaca, NY 14853, USA; 4 Howard Hughes Medical Institute, Department of Chemistry and Chemical Biology, Department of Molecular Biology and Genetics, Cornell University, Ithaca, NY 14853, USA; 5 These authors contributed equally; 6 Lead contact

## Abstract

Nucleotide oligomerization domain (NOD)-like receptor protein 3 (NLRP3) inflammasome hyperactivation contributes to many human chronic inflammatory diseases, and understanding how NLRP3 inflammasome is regulated can provide strategies to treat inflammatory diseases. Here, we demonstrate that NLRP3 Cys126 is palmitoylated by zinc finger DHHC-type palmitoyl transferase 7 (ZDHHC7), which is critical for NLRP3-mediated inflammasome activation. Perturbing NLRP3 Cys126 palmitoylation by ZDHHC7 knockout, pharmacological inhibition, or modification site mutation diminishes NLRP3 activation in macrophages. Furthermore, Cys126 palmitoylation is vital for inflammasome activation *in vivo*. Mechanistically, ZDHHC7-mediated NLRP3 Cys126 palmitoylation promotes resting NLRP3 localizing on the *trans*-Golgi network (TGN) and activated NLRP3 on the dispersed TGN, which is indispensable for recruitment and oligomerization of the adaptor ASC (apoptosis-associated speck-like protein containing a CARD). The activation of NLRP3 by ZDHHC7 is different from the termination effect mediated by ZDHHC12, highlighting versatile regulatory roles of *S*-palmitoylation. Our study identifies an important regulatory mechanism of NLRP3 activation that suggests targeting ZDHHC7 or the NLRP3 Cys126 residue as a potential therapeutic strategy to treat NLRP3-related human disorders.

## In brief

Yu et al. demonstrate that NLRP3 Cys126 can be palmitoylated by the palmitoyl transferase ZDHHC7 in macrophages, which is important for NLRP3 *trans*-Golgi network localization and the recruitment of the adaptor protein ASC, consequently promoting inflammasome assembly and accelerating the inflammatory responses in endotoxic shock and peritonitis *in vivo*.

## Graphical abstract

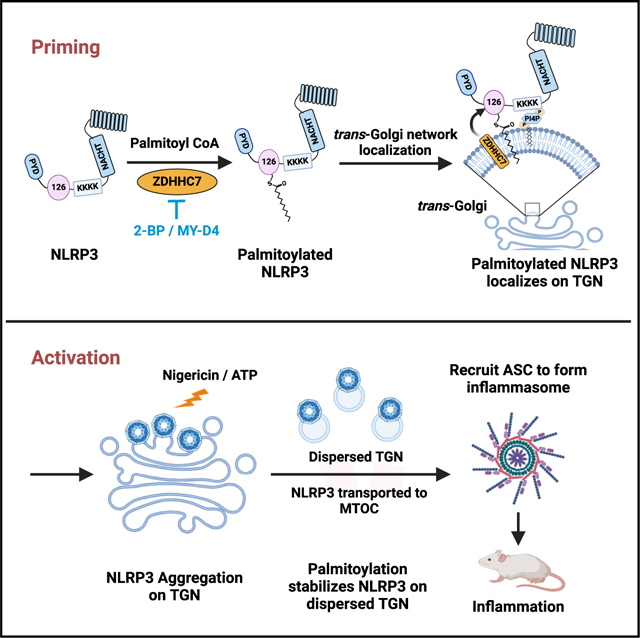

## INTRODUCTION

Macrophages play important roles in defending hosts from pathogen infection or sensing intracellular dangerous molecules. The pattern recognition receptors (PRRs) in macrophages recognize diverse pathogen-associated molecular patterns (PAMPs)^1^ or damage-associated molecular patterns (DAMPs)^2^ and promote pro-inflammatory responses by secreting cytokines such as interleukin-1β (IL-1β). NOD-like receptor protein 3 (NLRP3) is a cytosolic PRR that responds to multiple PAMPs/DAMPs and forms a multicomponent complex called the NLRP3 inflammasome to promote inflammation.^3^ The activation of the NLRP3 inflammasome involves a priming step and an activation step.^4^ In the priming step, lipopolysaccharides (LPSs) or other signals are recognized by cell-surface PRRs and activate nuclear factor κB (NF-κB) to induce expression of NLRP3, pro-IL-1β, and pro-IL-18.^3,4^ During the activation step, multiple PAMPs or DAMPs, including the bacterial pore-forming toxin nigericin and extracellular adenosine triphosphate (ATP), induce potassium efflux and mitochondrial reactive oxygen species production, which activate NLRP3.^3–5^ Activated NLRP3 recruits ASC and NEK7 to form the inflammasome complex.^5,6^ The NLRP3 inflammasome complex then recruits and activates caspase-1, which cleaves pro-IL-1β and pro-IL-18 to form mature IL-1β and IL-18.^4,5^ Activated caspase-1 also cleaves gasdermin D (GSDMD).^7,8^ The released N-terminal half of GSDMD is inserted into the plasma membrane and forms pores^9,10^ to release mature IL-1β and IL-18^7,8,10^ as well as to induce cell death (pyroptosis),^8,9^ which contributes to the release of more intracellular components, including lactate dehydrogenase (LDH)^7,10^ and high-mobility group box 1 (HMGB1).^11^

NLRP3 is involved in many inflammatory and autoimmune diseases.^4^ Gain-of-function NLRP3 mutations are relevant with cryopyrin-associated periodic syndromes (CAPSs),^12–14^ a group of rare heritable autoinflammatory diseases that includes familial cold auto-inflammatory syndrome (FCAS); chronic infantile neurological, cutaneous, and articular (CINCA) syndrome; and Muckle-Wells syndrome (MWS).^15^ Hyperactivation of the NLRP3 inflammasome is associated with neurodegenerative diseases,^16^ gout,^17^ diabetes,^18^ pathogen infection-induced septic shock,^19,20^ and cytokine storm.^21,22^ Therefore, accurate regulation of NLRP3 activation is critically important. Recent work shows that resting NLRP3 forms double-ring cages and is localized on the *trans*-Golgi network (TGN); the TGN localization is crucial for NLRP3 activation when responding to danger signals.^23,24^ However, what regulates NLRP3 TGN localization is unknown. Understanding how NLRP3 localization is governed will provide important insight into the precise regulation of the NLRP3 inflammasome, providing potential targets for treating NLRP3-related human disorders.

*S*-palmitoylation is the addition of a long-chain fatty acyl group, typically the 16-carbon palmitoyl group, to cysteine residues of target proteins.^25–27^ It is catalyzed by a group of palmitoyl transferases, ZDHHCs (ZDHHC1–ZDHHC23 in humans) that contain a conserved DHHC (Asp-His-His-Cys) cysteine-rich domain that is important for the enzymatic activity.^25,28^ A few proteins involved in immune signaling have been identified to be modified by *S*-palmitoylation, such as signal transducer and activator of transcription 3 (STAT3),^26^ NOD1/2,^29,30^ stimulator of interferon genes protein (STING),^31^ and programmed cell death ligand 1 (PD-L1). ^32^ Typically, protein *S*-palmitoylation promotes membrane targeting of otherwise soluble proteins through its hydrophobic chain,^25,28,33^ which, in turn, affects protein-protein interaction and signal transduction.^25,33^ Here, we report that NLRP3 can be *S*-palmitoylated by the palmitoyl acyltransferase ZDHHC7 on Cys126 in macrophages, which is important for the TGN localization of resting NLRP3 and the dispersed TGN (dTGN) localization of activated NLRP3, consequently promoting ASC recruitment and downstream NLRP3 inflammasome activation and ultimately accelerating endotoxic shock and peritonitis *in vivo*.

## RESULTS

### NLRP3 is *S*-palmitoylated by ZDHHC7 in macrophages

**Figure 1.**
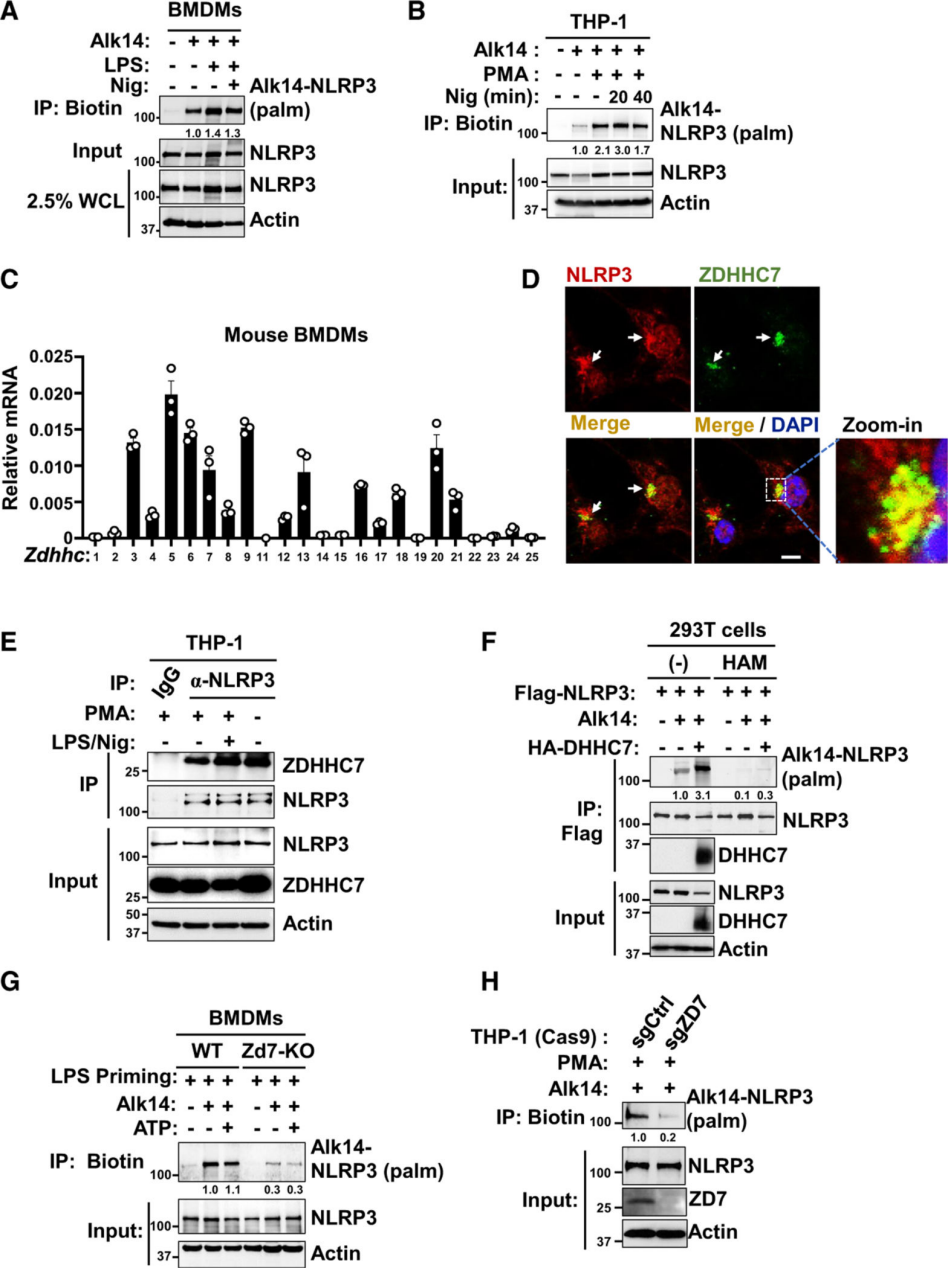
NLRP3 is *S*-palmitoylated by ZDHHC7 in macrophages (A) Palmitoylation of NLRP3 in BMDMs by Alk14 labeling and click chemistry assay. The NLRP3 palmitoylation level was quantified and normalized to NLRP3 protein levels in the input samples. (B) NLRP3 palmitoylation in PMA-differentiated THP-1 cells treated with Alk14, LPS, and nigericin. (C) Relative mRNA of *Zdhhc* genes in BMDMs determined by quantitative real-time qPCR; the mRNA level was normalized to β-actin. Data are represented as mean ± SEM. (D) Representative confocal images of NLRP3 and ZDHHC7 in LPS-primed BMDMs; the nucleus was stained with DAPI. Scale bar: 5 μm. (E) THP-1 cells were differentiated with PMA and then treated with LPS and nigericin. Cell lysates were collected for IP to detect endogenous NLRP3-ZDHHC7 association. (F) Palmitoylation of FLAG-NLRP3 in HEK293Tcells with ZDHHC7 expression was detected using Alk14 labeling and in-gel fluorescence. Hydroxylamine (HAM) treatment was used to confirm *S*-palmitoylation, which is HAM sensitive; the NLRP3 palmitoylation level was normalized to NLRP3 protein levels in the IP samples. (G) NLRP3 palmitoylation in WT and *Zdhhc7* KO BMDMs, determined with Alk14 labeling. Cells were primed with LPS and activated with ATP as indicated. (H) Palmitoylation of NLRP3 was determined in WT and *ZDHHC7* KO THP-1 cells. See also Figures S1–S4.

Protein *S*-palmitoylation is considered to have important roles in inflammatory responses.^25^ To investigate NLRP3 palmitoylation, we incubated mouse bone marrow-derived macrophages (BMDMs) with a clickable palmitate analog, Alkyne 14 (Alk14), a chemical reporter for palmitoylation,^26,34,35^ performed click chemistry to conjugate Alk14-labeled proteins with biotin, and determined the palmitoylation levels by streptavidin pull-down and immunoblot. The results showed that NLRP3 was labeled with Alk14, suggesting that endogenous NLRP3 is palmitoylated in BMDMs (Figure 1A). Similarly, the palmitoylation of NLRP3 was also detectable in a phorbol 12-myristate 13-acetate (PMA)-differentiated human macrophage cell line, THP-1 (Figure 1B), suggesting that NLRP3 palmitoylation occurred in both mouse and human macrophages.

To identify which ZDHHC protein could mediate NLRP3 modification, we determined the expression of all *Zdhhc* genes (*Zdhhc1–Zdhhc9* and *Zdhhc11–Zdhhc25*) in BMDMs (Figure 1C) and analyzed the expression level of all the *ZDHHC* genes (*ZDHHC1–ZDHHC9*, *ZDHHC11*, *ZDHHC11b*, and *ZDHHC12–ZDHHC24*) in human classical monocytes from a public database^36^ (The Human Protein Atlas; Figure S1A). We found that several *Zdhhc* genes showed a predominant expression level in BMDMs, including *Zdhhc7* (Figure 1C). In human CD14^high^ CD16^−^ classical monocytes from a cohort of 91 healthy individuals, *ZDHHC7* showed predominant expression compared with other *ZDHHC* genes (Figure S1A), suggesting that *ZDHHC7* potentially has an important role in macrophages.

To investigate whether ZDHHC7 could mediate NLRP3 palmitoylation in macrophages, we first determined whether ZDHHC7 interacts with NLRP3. Through immunofluorescence staining, we found that NLRP3 co-localized with ZDHHC7 in the resting state in LPS-primed BMDMs (Figure 1D). A fluorescence resonance energy transfer (FRET) assay further confirmed the interaction between NLRP3 and ZDHHC7 in LPS-primed BMDMs (Figure S1B). Additionally, a co-immunoprecipitation (coIP) assay showed NLRP3 IP could pull down ZDHHC7 in THP-1 cells in resting, PMA-differentiated, or nigericin-activated states (Figure 1E). These data collectively suggest that NLRP3 associates with ZDHHC7 in macrophages.

We next co-expressed NLRP3 and ZDHHC7 in human embryonic kidney (HEK) 293T cells to see whether ZDHHC7 expression could increase NLRP3 palmitoylation. We found that ZDHHC7 expression increased NLRP3 palmitoylation and that hydroxylamine abolished ZDHHC7-mediated NLRP3 palmitoylation, suggesting that the palmitoylation of NLRP3 is *S*-palmitoylation on cysteine residues (Figure 1F). Furthermore, deletion of *ZDHHC7* reduced the palmitoylation of NLRP3 expressed in HEK293T cells (Figure S1C), and the enzymatically inactive mutant of ZDHHC7 (ZDHHS7) was unable to restore NLRP3 palmitoylation in *ZDHHC7*-deleted HEK293T cells, in contrast to wild-type ZDHHC7 (Figure S1C), confirming that the enzymatic activity of ZDHHC7 was essential for NLRP3 palmitoylation. Last, to further confirm that NLRP3 *S*-palmitoylation was induced by ZDHHC7 in macrophages, we isolated BMDMs from *Zdhhc7* knockout mouse, generated *ZDHHC7* knockout THP-1 cells, and determined NLRP3 *S*-palmitoylation using Alk14 labeling. The results showed that *Zdhhc7* deletion in mouse BMDMs and *ZDHHC7* deletion in human THP-1 cells dramatically diminished NLRP3 *S*-palmitoylation (Figures 1G and 1H).

To investigate whether other ZDHHC proteins could mediate NLRP3 *S*-palmitoylation, we also expressed other ZDHHCs in HEK293T cells and performed NLRP3 Alk14 labeling. While several ZDHHC proteins (ZDHHC3, ZDHHC6, ZDHHC7, and ZDHHC9) could promote NLRP3 *S*-palmitoylation, ZDHHC7 exhibited the strongest activity (Figures S1D and S1E). To check whether ZDHHC3, ZDHHC6, and ZDHHC9, which exhibited potential activity for NLRP3 *S*-palmitoylation in HEK293T cells and high expression levels in BMDMs, could mediate NLRP3 *S*-palmitoylation in macrophages, we isolated BMDMs from *Zdhhc3*, *Zdhhc6*, or *Zdhhc9* knockout mice and found that the knockout did not affect NLRP3 *S*-palmitoylation (Figures S1F and S1G). Taken together, these data strongly demonstrate that NLRP3 is modified with *S*-palmitoylation mainly by ZDHHC7 in macrophages.

### ZDHHC7-catalyzed *S*-palmitoylation is important for NLRP3 inflammasome activation

To investigate whether ZDHHC7-mediated *S*-palmitoylation regulates inflammasome activation in macrophages, we first studied NLRP3 inflammasome regulation by 2-bromopalmitate (2-BP), a pan-ZDHHC inhibitor, in BMDMs. 2-BP potently repressed NLRP3 *S*-palmitoylation in BMDMs (Figure S2A) but did not affect protein levels of NLRP3, pro-caspase-1, or GSDMD (Figures S2A and S2B), suggesting that 2-BP treatment did not regulate the priming step. In contrast, 2-BP potently suppressed caspase-1 and GSDMD cleavage ase well as IL-1β secretion in BMDMs activated by nigericin (Figures S2B and S2C), which is the typical readout of NLRP3 inflammasome activation, suggesting that 2-BP inhibited the activation step of the NLRP3 inflammasome. Additionally, MY-D4, a more recent improved inhibitor of ZDHHC enzymes,^37,38^ also exhibited strong inhibition of caspase-1 and GSDMD cleavage (Figure S2D) in BMDMs, further confirming that ZDHHC inhibition could repress NLRP3 inflammasome activation. We also evaluated the effect of ZDHHC inhibitors on inflammasome activation in human macrophages, including THP-1 and human peripheral blood mononuclear cell (PBMC)-derived primary macrophages. 2-BP treatment inhibited GSDMD cleavage and IL-1β secretion in both PMA-differentiated THP-1 cells and human primary macrophages (Figures S2E and S2F). Another inhibitor, MY-D4, also showed potent inhibition of GSDMD cleavage and IL-1β secretion in THP-1 cells (Figures S2G and S2H). The data collectively demonstrate that NLRP3 *S*-palmitoylation is important for NLRP3 inflammasome activation in both mouse and human macrophages and can be suppressed by ZDHHC inhibitors.

Next, we confirmed whether ZDHHC7-mediated NLRP3 *S*-palmitoylation is important for NLRP3 inflammasome activation. We first evaluated whether ZDHHC7 regulates the LPS priming process. We found that *Zdhhc7* knockout (KO) did not affect LPS-induced p65 phosphorylation (Figure S3A), suggesting that ZDHHC7 does not regulate the activation of NF-κB, the major transcription factor that mediates the expression of inflammasome components during LPS priming. In agreement with this finding, *Zdhhc7* KO did not affect the LPS-induced mRNA expression of *IL-1b*/*Il-18* (Figure S3B) or protein levels of NLRP3, pro-caspase-1, or full-length GSDMD (Figure S3C), further validating that ZDHHC7 did not regulate the priming step, which is consistent with the effect of 2-BP described above. To find out whether ZDHHC7 affects the activation step of NLRP3 inflammasome, we determined the cleavage of caspase-1 and GSDMD and IL-1β secretion. *Zdhhc7* KO potently inhibited caspase-1 and GSDMD cleavage (Figure S3C) and IL-1β secretion (Figure S3D) after inflammasome activation in BMDMs, suggesting that ZDHHC7 is important for the activation process of the NLRP3 inflammasome in mouse macrophages. To verify this conclusion in human macrophages, we knocked out *ZDHHC7* in human THP-1 cells and found that *ZDHHC7* KO inhibited GSDMD cleavage and IL-1β secretion in THP-1 cells (Figures S3E and S3F). Additionally, as 2-BP has been reported to have off targets,^39^ to further verify that the effect of 2-BP in regulating the NLRP3 inflammasome was through ZDHHC7, we synthesized a 2-BP-Alk probe that can be used to label proteins targeted by 2-BP (Figure S3G). Using 2-BP-Alk incubation, conjugation of biotin-azide, and streptavidin pulldown, we verified that 2-BP-Alk could covalently bind to ZDHHC7 in inflammasome-activated BMDMs (Figure S3H). Importantly, 2-BP treatment did not further suppress GSDMD cleavage or pyroptosis (LDH release) in *Zdhhc7* KO BMDMs activated with nigericin (Figures S3I and S3J), suggesting that 2-BP inhibits NLRP3 inflammasome activation mainly through ZDHHC7. Taken together, these data collectively indicated that ZDHHC7-catalyzed *S*-palmitoylation is important for NLRP3 inflammasome activation in mouse and human macrophages.

In addition to the NLRP3 inflammasome, several other inflammasome complexes that respond to diverse danger signals have been studied in macrophages.^40^ Non-canonical inflammasome is triggered by cytosolic LPS of invasive gram-negative bacteria, which activates and oligomerizes caspase-4/5/11 to cleave GSDMD and induce pyroptosis.^41^ Absent in melanoma 2 (AIM2) senses double-stranded DNA from microbial pathogens or host cellular damage and recruits ASC to assemble the inflammasome complex, which activates caspase-1 and leads to pyroptosis and cytokine secretion.^42–44^ To investigate whether ZDHHC7 regulates this NLRP3-independent inflammasome activation, we determined GSDMD cleavage and pyroptosis (LDH release) in wild-type (WT) and *Zdhhc7*-deleted BMDMs under non-canonical or AIM2-mediated inflammasome conditions. We found that *Zdhhc7* deletion did not affect non-canonical-inflammasome-induced GSDMD cleavage or pyroptosis in BMDMs (Figures S4A and S4B). *Zdhhc7* deletion also did not affect GSDMD cleavage induced by the AIM2 inflammasome (Figure S4C). Thus, ZDHHC7 does not regulate non-canonical or AIM2 inflammasome activation.

Last, to check whether ZDHHC3, ZDHHC6, or ZDHHC9, which exhibited high expression in BMDMs, may regulate NLRP3 activation in macrophages, we also isolated BMDMs from *Zdhhc3*, *Zdhhc6*, or *Zdhhc9* KO mice and found that none of them affected GSDMD cleavage induced by ATP or nigericin (Figures S4D–S4F), suggesting that these ZDHHC proteins are not involved in NLRP3 activation.

### NLRP3 was *S*-palmitoylated on Cys126 by ZDHHC7

Thus far, our data demonstrated that ZDHHC7 can palmitoylate NLRP3 and promote NLRP3 inflammasome activation. However, whether the effect of ZDHHC7 on NLRP3 inflammasome activation is through NLRP3 *S*-palmitoylation or through other substrate proteins was not clear. To address this, we sought to identify the modified cysteine(s) on NLRP3 by ZDHHC7. Once the modified cysteine is identified, the cysteine mutant can be used to further validate that ZDHHC7’s effect on NLRP3 inflammasome activation is through NLRP3 palmitoylation.

**Figure 2.**
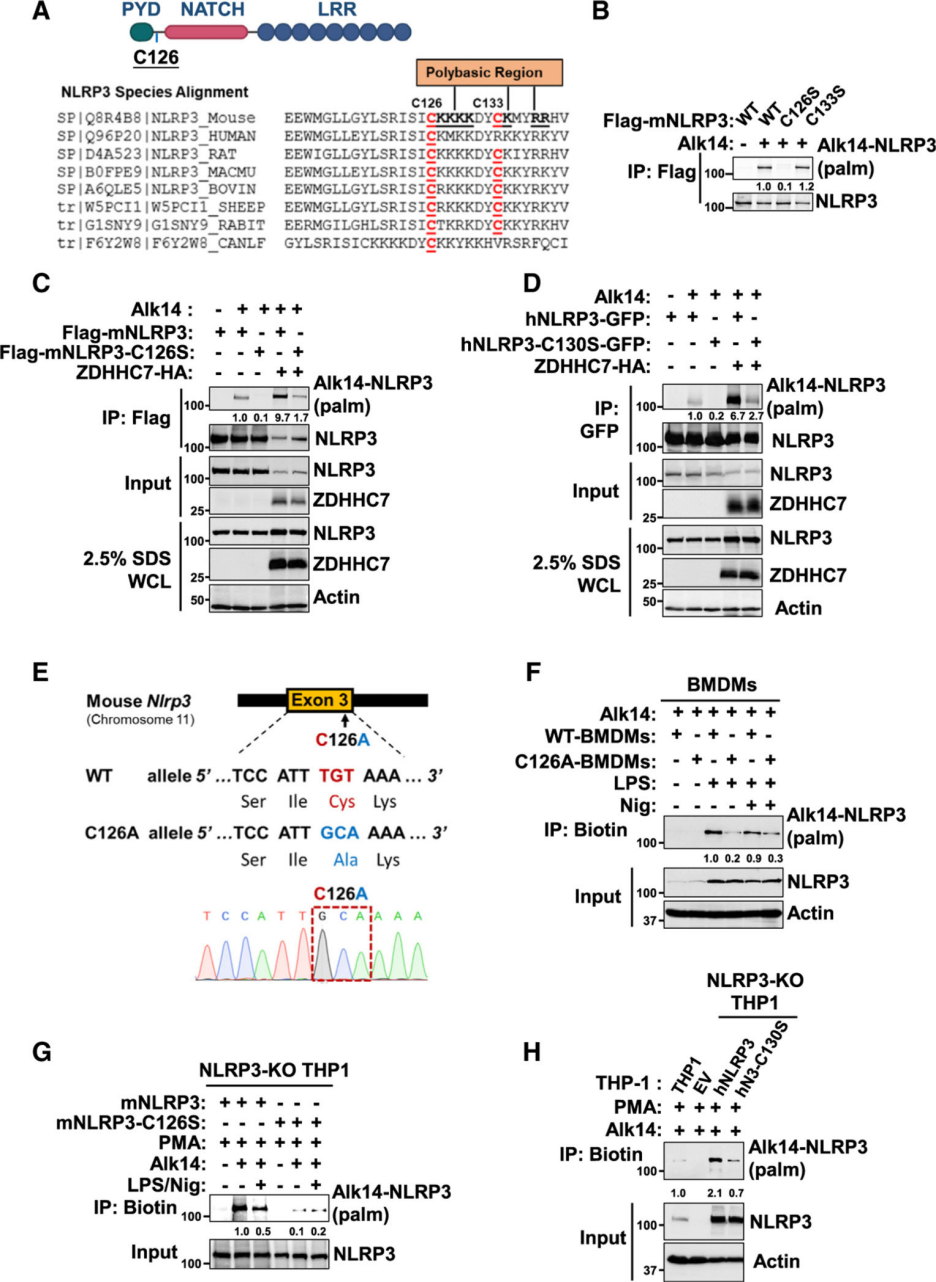
NLRP3 is *S*-palmitoylated on Cys126 by ZDHHC7 (A) Schematic of NLRP3 domains, showing the location of Cys126 and sequence alignment of NLRP3 from different species. (B) Palmitoylation of mouse NLRP3 WT and cysteine-to-serine mutants (CS) in HEK293T cells, determined with Alk14 labeling. (C and D) Palmitoylation of the mouse (C) and human (D) NLRP3 WT and cysteine-to-serine mutant (C126S of mouse NLRP3, C130S of human NLRP3) in HEK293T cells. (E) The generation of the CRISPR-edited NLRP3 C126A mutant (top) and sequencing verification (bottom). (F) Palmitoylation of NLRP3 in WT and C126ABMDMs by Alk14 labeling. (G) Palmitoylation of mouse NLRP3 WT and C126S that were reconstituted stably in NLRP3-deleted THP-1 cells. (H) Palmitoylation of human NLRP3 WT and C130S that were reconstituted stably in NLRP3-deleted THP-1 cells.

Recent work has reported that resting-state NLRP3 localizes to the TGN by ionic bonding between its conserved polybasic region and the negatively charged phosphatidylinositol 4-phosphates (PtdIns4P) on the TGN.^23,24,45^ We hypothesize that ZDHHC7 may palmitoylate NLRP3 and promote its TGN localization. Therefore, we screened the cysteine residues on NLRP3 and found that two cysteines, Cys126 and Cys133, are near the polybasic region (Figure 2A). Cys126 (human Cys130) is conserved in all NLRP3s examined while Cys133 is not. We mutated each of them to serine and performed Alk14 labeling. Mutating Cys126 to serine (C126S), but not Cys133 to serine (C133S), dramatically decreased the *S*-palmitoylation level of NLRP3 (Figure 2B). Additionally, the C126S mutant of mouse NLRP3 or the C130S mutant of human NLRP3 dramatically decreased ZDHHC7-promoted NLRP3 *S*-palmitoylation in HEK293T cells (Figure 2C and 2D), suggesting that Cys126 (human Cys130) is the major site of NLRP3 *S*-palmitoylation mediated by ZDHHC7.

To verify that NLRP3 Cys126 is the major physiological *S*-palmitoylation site in macrophages, we generated C126A *Nlrp3* gene-edited mice, with the TGT of *Nlrp3* alleles in exon 3, encoding Cys126, mutated to the alanine-encoded sequence GCA (TGT>GCA; Figure 2E), and determined NLRP3 *S*-palmitoylation in BMDMs. We found that the C126A mutant dramatically diminished the S-palmitoylation of NLRP3 in both LPS-primed and nigericin-activated BMDMs (Figure 2F), confirming that Cys126 is the major *S*-palmitoylation site in mouse primary macrophages. To investigate whether the corresponding Cys130 in human NLRP3 is the major palmitoylation site in human macrophages, we depleted endogenous NLRP3 in THP-1 cells and then reconstituted with either the WT or cysteine mutant NLRP3. Both mouse C126S and human C130S NLRP3 mutants had dramatically decreased modification levels in THP-1 cells compared with WT NLRP3 (Figures 2G and 2H). Therefore, these data collectively indicate that Cys126 is the major site of NLRP3 *S*-palmitoylation mediated by ZDHHC7 in macrophages.

### NLRP3 Cys126 palmitoylation by ZDHHC7 is crucial for NLRP3 inflammasome activation in macrophages

**Figure 3.**
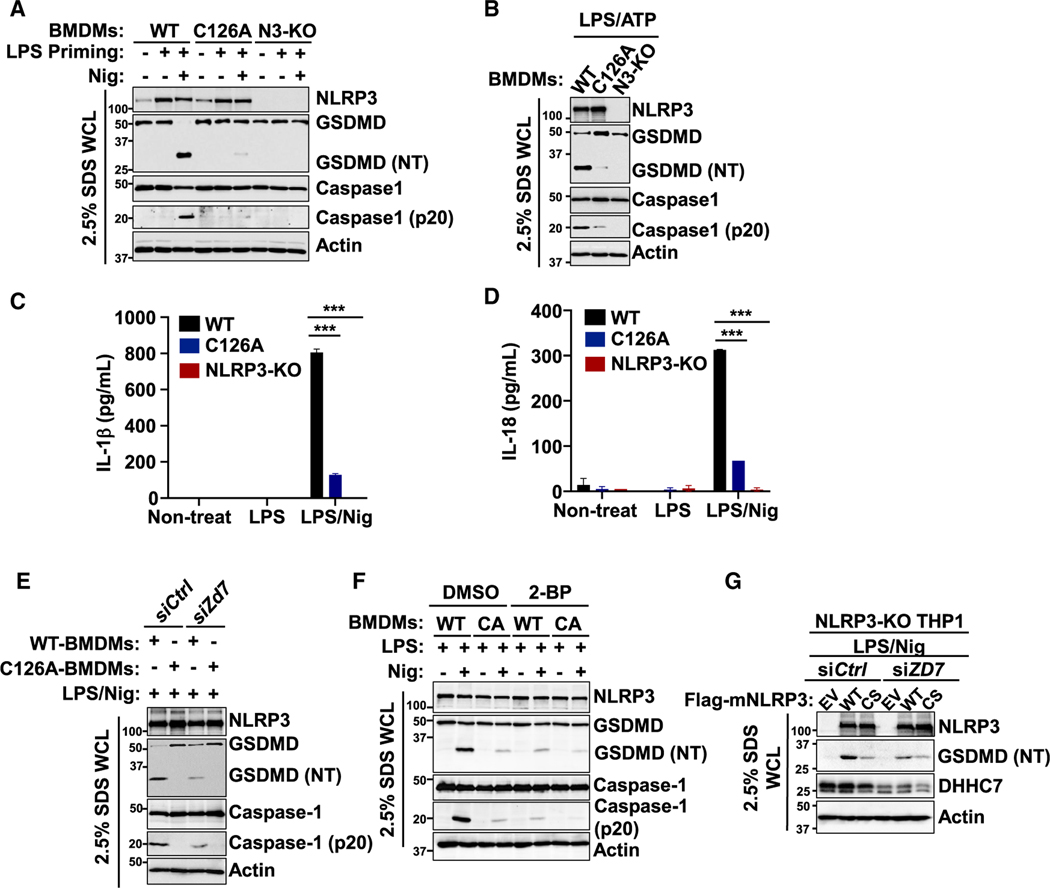
ZDHHC7-mediated NLRP3 Cys126 palmitoylation is important for NLRP3 inflammasome activation in macrophages (A and B) Immunoblot analysis of NLRP3, caspase-1, GSDMD, cleaved caspase-1 (p20), and cleaved GSDMD (NT) in whole-cell lysate (WCL) of NLRP3 WT, C126A, and KO (N3-KO) BMDMs that were primed with LPS and activated with nigericin (Nig, A) or ATP (B). Cells were lysed with 2.5% SDS lysis buffer. (C and D) Levels of IL-1β (C) and IL-18 (D) in the cell culture medium of BMDMs in (A). (E) Immunoblot analysis in WCL of NLRP3 WT and C126A BMDMs that had *Zdhhc7* knocked down (si*Zd7*), primed with LPS, and activated with Nig. (F) Immunoblot analysis in WCL of NLRP3 WT and C126A (CA) BMDMs that were primed with LPS and treated with DMSO or 2-BP before activation with Nig. (G) Immunoblot analysis in NLRP3-deleted THP-1 cells that were reconstituted with WT or C126S (CS) NLRP3 and had *ZDHHC7* knocked down (si*ZD7*). Data with error bars are mean ± SEM. *p < 0.05, **p < 0.01, ***p < 0.001, as determined by unpaired Student’s t test. See also Figure S5.

To confirm the importance of Cys126 palmitoylation for NLRP3 inflammasome activation, we determined NLRP3 inflammasome activation in BMDMs differentiated from WT, CRISPR-edited C126A, and *Nlrp3* KO mice. We found that caspase-1 and GSDMD cleavage (Figures 3A and 3B) as well as IL-1β and IL-18 secretion in C126A BMDMs were dramatically inhibited compared with in WT BMDMs (Figures 3C and 3D). The effect of the C126A mutation was close to that in *Nlrp3* KO BMDMs (Figures 3A–3D). Furthermore, we also compared the effect of *Zdhhc7* knockdown on inflammasome activation with NLRP3 WT and the C126A mutant in BMDMs. *Zdhhc7* knockdown suppressed caspase-1 and GSDMD cleavage in NLRP3 WT BMDMs but not the NLRP3 C126A mutant BMDMs (Figure 3E and S5A), supporting the theory that ZDHHC7’s effect on NLRP3 inflammasome activation is mainly through C126 palmitoylation. Similarly, 2-BP treatment significantly decreased caspase-1 and GSDMD cleavage in WT BMDMs but not in C126A mutant BMDMs (Figure 3F). Therefore, Cys126 palmitoylation is crucial for NLRP3 inflammasome activation in mouse primary macrophages, and the effect of ZDHHC7 on NLRP3 inflammasome activation occurs mainly through Cys126 palmitoylation.

We also investigated whether NLRP3 Cys126 palmitoylation has a vital function in human macrophages. We introduced WT and C126S NLRP3 in THP-1 cells where the endogenous NLRP3 is deleted. WT NLRP3 reconstitution restored GSDMD cleavage and IL-1β secretion, but C126S reconstitution did not (Figures S5B–S5D). Moreover, *ZDHHC7* knockdown suppressed GSDMD cleavage induced by WT NLRP3 but not by the C126S mutant (Figure 3G), suggesting that the effect of ZDHHC7 on NLRP3 inflammasome activation occurs mainly through Cys126 palmitoylation. Similarly, 2-BP treatment decreased GSDMD cleavage and IL-1β secretion induced by WT NLRP3 but not by the C126S mutant in the THP-1 cells that were depleted of endogenous NLRP3 (Figures S5E and S5F). Taken together, these data support the theory that ZDHHC7-mediated NLRP3 Cys126 *S*-palmitoylation is important for NLRP3 inflammasome activation in human macrophages.

### ZDHHC7-catalyzed Cys126 palmitoylation promotes NLRP3 TGN localization

**Figure 4.**
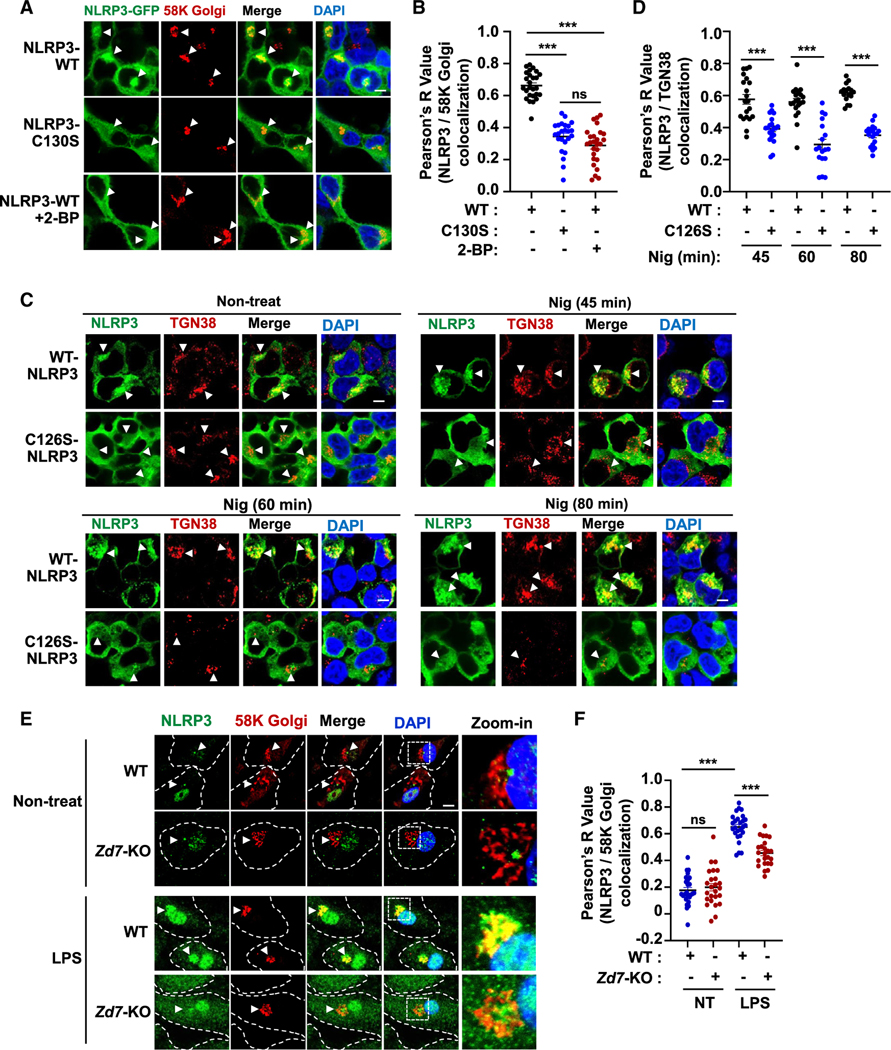
NLRP3 Cys126 palmitoylation promotes NLRP3 localization on the TGN (A) Representative confocal images of GFP-tagged human NLRP3 WT or C130S expressed in HEK293T cells that were treated with DMSO or 2-BP. NLRP3-GFP is shown in green, the TGN was stained with anti-58K Golgi (red), and the nucleus was stained with DAPI (blue). Scale bar: 5 μm. (B) Quantification of images in (A) by Pearson’s correlation coefficient using ImageJ. (C) Confocal images of the WT and CS mutant of mouse NLRP3-FLAG in HEK293T cells that were treated with Nig for the indicated times. NLRP3 was stained with anti-FLAG (green), the TGN was stained with anti-TGN38 (red), and the nucleus was stained with DAPI (blue). Scale bar: 5 μm. (D) Quantification of images in (C) by Pearson’s correlation coefficient using ImageJ. (E) Representative confocal images for endogenous NLRP3 in WT and *Zdhhc7* KO (*Zd7*-KO) BMDMs. NLRP3 was stained green, the TGN was stained with anti-58K Golgi (red), and the nucleus was stained with DAPI (blue). Scale bar: 5 μm. (F) Quantification of images in (E) by Pearson’s correlation coefficient. Data with error bars are mean ± SEM. *p < 0.05, **p < 0.01, ***p < 0.001, as determined by unpaired Student’s t test. See also Figure S6.

We next investigated how Cys126 palmitoylation promotes NLRP3 inflammasome activation. *S*-palmitoylation promotes protein anchoring into the membrane lipid bilayer, which, in turn, regulates protein subcellular localization and protein-protein interactions.^26,29,31^ For instance, ZDHHC7-catalyzed STAT3 *S*-palmitoylation mediates its membrane localization and JAK2-STAT3 interaction, which, in turn, promotes STAT3 activation.^26^ Recent work elucidated that resting NLRP3 is mainly membrane bound and forms oligomeric double-ring cages on the TGN, which is necessary for NLRP3 inflammasome activation.^23,24,46^ Therefore, we investigated whether NLRP3 *S*-palmitoylation promotes its TGN localization. By expressing NLRP3 fused with GFP in HEK293T cells, we found that resting NLRP3 indeed co-localized with the TGN markers 58K Golgi (Figures 4A and 4B) or TGN38 (Figures 4C and S6A). Furthermore, 2-BP treatment, NLRP3 *S*-palmitoylation site mutants, or *ZDHHC7* deletion significantly inhibited resting NLRP3 localization on the TGN (Figures 4A, 4B, and S6A). Therefore, NLRP3 Cys126 *S*-palmitoylation is important for resting NLRP3 TGN localization.

After NLRP3 is activated, the TGN will be disrupted to form the dTGN vesicle, which will carry NLRP3 to the microtubular organization center (MTOC),^47^ where NLRP3 will recruit NEK7 and the adaptor protein ASC to form the inflammasome complex.^47,48^ Our data showed that nigericin-activated NLRP3 was localized on the dTGN (Figure 4C), which was consistent with previous reports,^23,47^ whereas C126S mutant abolished the NLRP3 dTGN localization (Figures 4C and 4D), indicating that NLRP3 Cys126 *S*-palmitoylation is also important for its dTGN localization after NLRP3 activation. Endogenous NLRP3 was localized on the TGN in the LPS-primed resting state, while *Zdhhc7* KO inhibited the endogenous NLRP3 TGN localization in BMDMs (Figures 4E and 4F). Interestingly, Golgi apparatus-localized ZDHHC7 was dispersed on the dTGN during inflammasome activation (Figure S6B), and NLRP3 continued to be co-localized with ZDHHC7 after NLRP3 activation in BMDMs (Figure S6C). These data strongly suggest that ZDHHC7-mediated NLRP3 Cys126 *S*-palmitoylation is necessary for NLRP3 TGN/dTGN localization.

### ZDHHC7-catalyzed Cys126 palmitoylation promotes ASC recruitment and inflammasome assembly after NLRP3 activation

**Figure 5.**
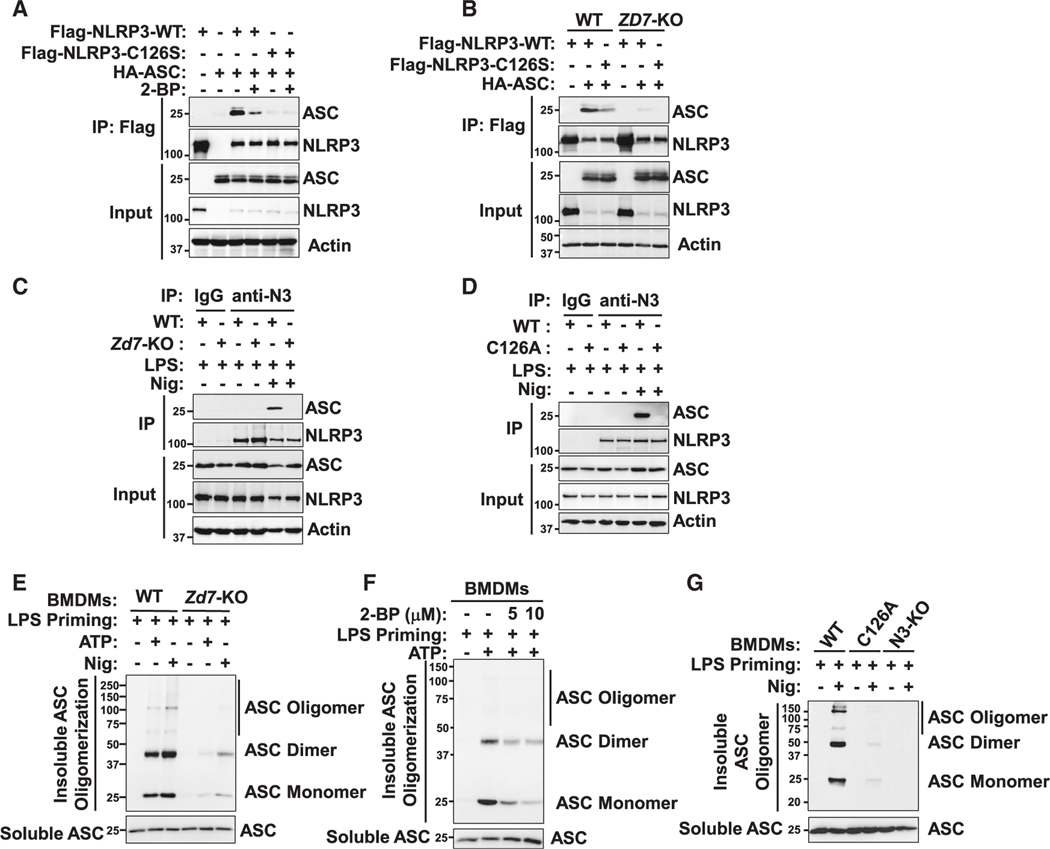
Cys126 palmitoylation promotes ASC recruitment and oligomerization after inflammasome activation (A) Immunoblot analysis of NLRP3 interaction with ASC expressed in HEK293T cells by coIP with DMSO or 2-BP treatment for 12 h. (B) NLRP3 interaction with ASC expressed in WT or *ZDHHC7* KO (*ZD7*-KO) HEK293T cells detected by coIP. (C and D) NLRP3 interaction with ASC in WT and *Zdhhc7* KO BMDMs (C) or WT and *Nlrp3* C126A BMDMs (D). Cells were primed with LPS and activated with Nig. IP was performed with anti-NLRP3 (anti-N3), and immunoblotting was done with anti-ASC and anti-NLRP3. (E–G) Immunoblot analysis of insoluble ASC and its oligomerization in ZDHHC7 WT or KO BMDMs (E), in BMDMs that were treated with DMSO or 2-BP (F), and in NLRP3 WT, C126A, or N3-KO BMDMs (G). Disuccinimidyl suberate (DSS) was used to cross-link ASC in BMDMs activated with ATP or Nig as indicated. See also Figure S6.

NLRP3 dTGN localization acts as an oligomerization scaffold for downstream adaptor ASC recruitment after inflammasome activation and trafficking to the MTOC.^23,24,49^ Thus, we studied whether ZDHHC7-mediated Cys126 palmitoylation would affect the recruitment of ASC. We performed coIP assays by expressing NLRP3 and ASC proteins in HEK293T cells and found that 2-BP treatment inhibited NLRP3 interaction with ASC in HEK293T cells (Figure 5A). NLRP3 C126S mutation also decreased the interaction of NLRP3 and ASC, while 2-BP treatment had no effect on the interaction between ASC and NLRP3 C126S (Figure 5A), suggesting that the effect of 2-BP on NLRP3-ASC interaction occurs mainly through inhibiting NLRP3 Cys126 palmitoylation. As expected, *ZDHHC7* KO in HEK293T cells strongly suppressed NLRP3 WT interaction with ASC (Figure 5B) but had little effect on NLRP3 C126S mutant interaction with ASC, suggesting that ZDHHC7 contributed to the recruitment of ASC through Cys126 palmitoylation. To further confirm this in a more physiological setting, we examined the effect of *Zdhhc7* KO, 2-BP treatment, or NLRP3 C126A mutation on the interaction of NLRP3 with ASC after inflammasome activation in macrophages. Both *Zdhhc7* KO and 2-BP decreased the interaction of NLRP3 with ASC (Figures 5C and S6D), and the NLRP3 C126A mutant had a dramatically decreased interaction with ASC in BMDMs compared with NLRP3 WT (Figure 5D). The data collectively demonstrated that ZDHHC7-promoted NLRP3 Cys126 palmitoylation was critical for ASC recruitment after NLRP3 activation in macrophages.

As ASC recruitment is important for inflammasome complex assembly, we evaluated the regulation of NLRP3 Cys126 palmitoylation on inflammasome complex formation, especially ASC oligomerization. Our data indicated that *Zdhhc7* KO, 2-BP treatment, or NLRP3 C126A mutation dramatically inhibited ASC oligomerization induced by NLRP3 activation in BMDMs (Figures 5E–5G), supporting the theory that ZDHHC7-mediated NLRP3 Cys126 palmitoylation is important for inflammasome complex assembly after NLRP3 activation. Similarly, we found that ASC oligomerization could be rescued by WT NLRP3 reconstitution in NLRP3 KO human THP-1 cells, but not by NLRP3 C126S mutant reconstitution (Figure S6E). Therefore, ZDHHC7-catalyzed Cys126 palmitoylation is important for ASC recruitment and inflammasome assembly after NLRP3 activation in macrophages.

### ZDHHC7-mediated Cys126 palmitoylation regulates NLRP3 inflammasome activation *in vivo*

**Figure 6.**
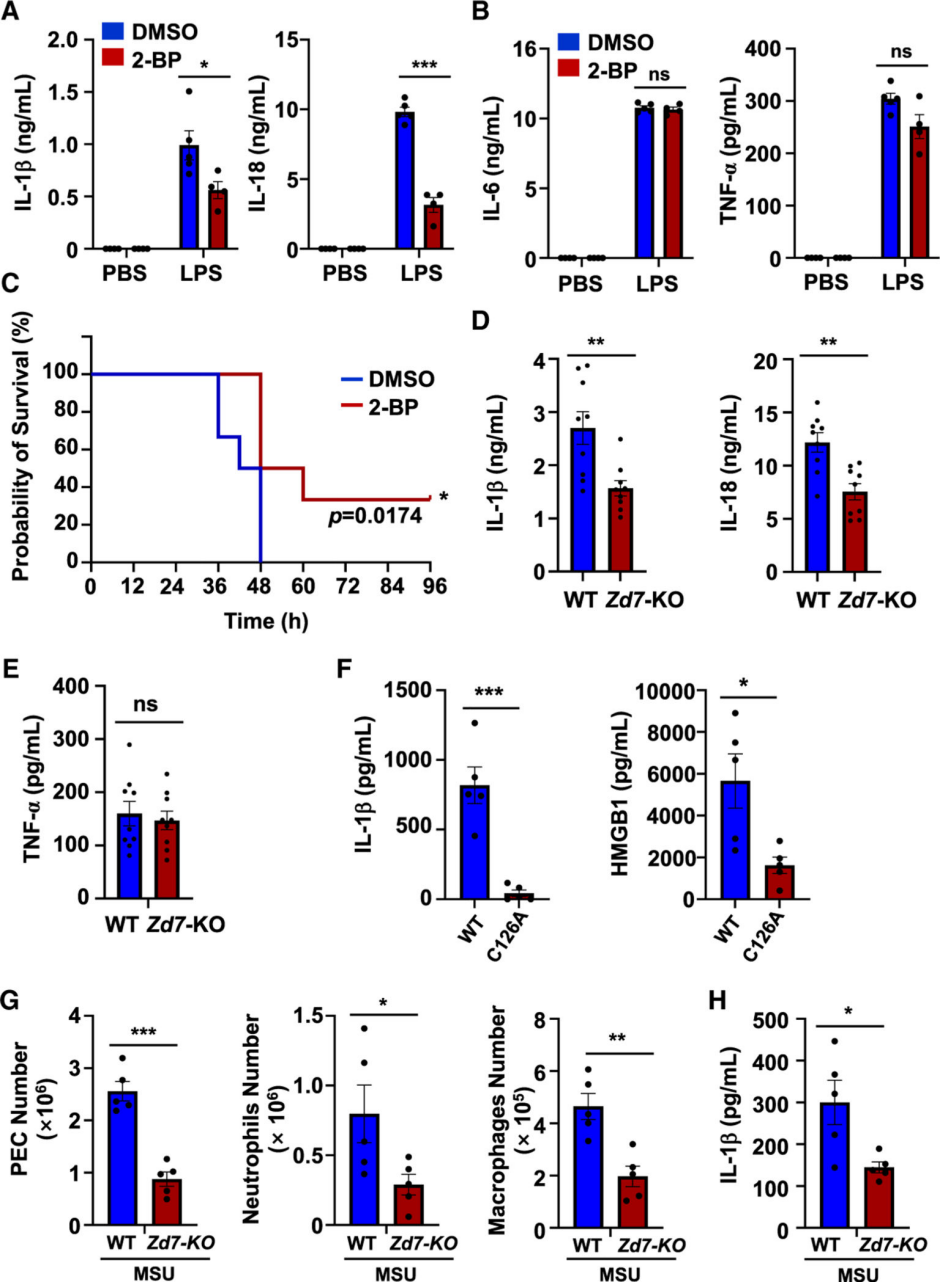
ZDHHC7-promoted Cys126 palmitoylation affects NLRP3 inflammasome activation *in vivo* (A and B) Mouse serum IL-1β and IL-18 (A) and IL-6 and TNF-α (B), measured by ELISA for WT mice with LPS-induced endotoxic shock after administration of DMSO or 2-BP (LPS, *n* = 5 or 4; PBS, *n* = 4). (C) Survival data of mice in response to LPS challenge after administration of DMSO or 2-BP (*n* = 6). The survival curve was statistically analyzed with log rank (Mantel-Cox) test. (D and E) Mouse serum IL-1β and IL-18 (D) and TNF-α (E), measured by ELISA for ZDHHC7 WT and KO (*Zd7*-KO) mice with endotoxic shock (*n* = 9). Mice were administered LPS for 8 h, and serum was collected for ELISA. (F) Serum IL-1β and HMGB1, measured by ELISA, for NLRP3 WT and C126A mice with endotoxic shock (*n* = 5). (G and H) Numbers of peritoneal exudate cells (PECs) and infiltrated neutrophils and macrophages in peritoneal lavage fluid (G) and ELISA of IL-1β in the serum (H) of ZDHHC7 WT and KO (*Zd7*-KO) mice that were injected intraperitoneally (i.p.) with LPS and MSU (*n* = 5). Data with error bars are mean ± SEM. *p < 0.05, **p < 0.01, ***p < 0.001, as determined by unpaired Student’s t test. See also Figure S7.

Next, we sought to test whether ZDHHC7-mediated NLRP3 Cys126 palmitoylation is also functioning *in vivo*. The LPS intraperitoneal injection-induced endotoxic shock and monosodium urate crystal (MSU)-induced peritonitis mouse models are widely used to assess NLRP3-dependent inflammasome responses *in vivo*.^47,50–52^ Therefore, we used these two models in mice to validate the regulation of NLRP3 *S*-palmitoylation *in vivo*. We found that 2-BP administration in mice inhibited LPS-induced IL-1β and IL-18 secretion (Figure 6A) but not tumor necrosis factor alpha (TNF-α) or IL-6 secretion (Figure 6B), which is mediated by NF-κB signaling. This suggests that 2-BP administration inhibits inflammasome activation *in vivo* but not NF-κB activation. Furthermore, 2-BP administration alleviated the mortality of endotoxic shock animals (Figure 6C). More importantly, *Zdhhc7* KO mice had lower IL-1β and IL-18 secretion (Figure 6D) and were protected against LPS-induced death (Figure S7A), while TNF-α secretion was similar in *Zdhhc7* KO and WT mice (Figure 6E). We also isolated peritoneal exudate cells (PECs) from these mice and found that *Zdhhc7* KO repressed NLRP3 palmitoylation levels in PEC (Figure S7B). Similarly, CRISPR-edited NLRP3 C126A also protected mice from endotoxic shock, with less circulating IL-1β and HMGB1 secretion (Figure 6F) and extended survival (Figure S7C). Compared with WT mice, NLRP3 palmitoylation was also decreased in the PECs of C126A mice with endotoxic shock (Figure S7D), indicating that NLRP3 is palmitoylated on Cys126 *in vivo*. Additionally, *Zdhhc7* KO mice were more resistant to MSU-induced peritonitis, with lower neutrophil and macrophage infiltration in peritoneal lavage fluid and diminished IL-1β levels in the serum of *Zdhhc7* KO mice (Figure 6G and 6H). All of these data were consistent with the cellular results and confirmed that NLRP3 *S*-palmitoylation regulated NLRP3 inflammasome activation *in vivo.*

### ZDHHC7-mediated NLRP3 activation is different from ZDHHC12-mediated inflammasome termination

**Figure 7.**
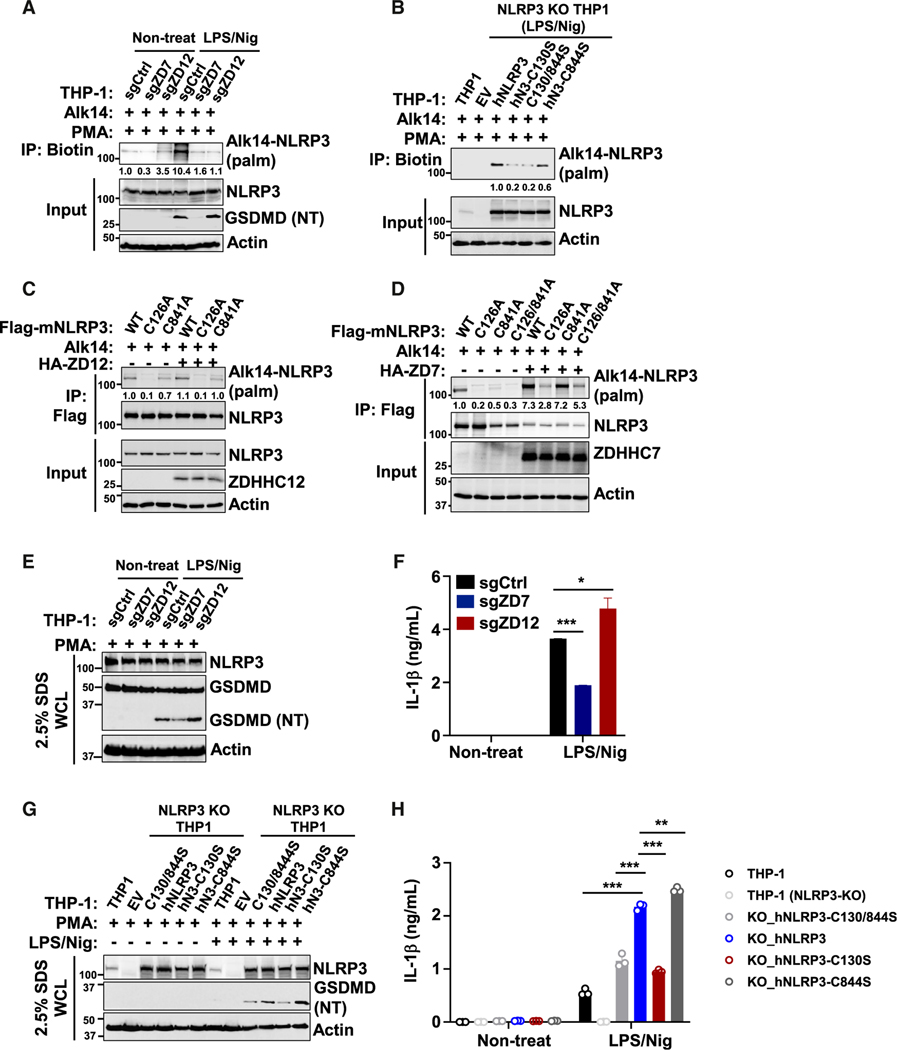
ZDHHC7 promotes NLRP3 activation, while ZDHHC12 terminates inflammasome signaling (A) Palmitoylation of NLRP3 in *ZDHHC7*-deleted (sgZD7), *ZDHHC12*-deleted (sgZD12), or control (sgCtrl) THP-1 cells differentiated with PMA, primed with LPS, and activated with Nig for 1 h. (B) Palmitoylation of NLRP3 in WT or NLRP3-KO THP-1 cells reconstituted with NLRP3 WT or cysteine-to-serine (CS) mutants. (C) Palmitoylation of NLRP3 WT or cysteine-to-alanine (CA) mutants expressed in HEK293T cells with ZDHHC12 (ZD12) co-expression. (D) Palmitoylation of NLRP3 WT or CA mutants in HEK293T cells with ZDHHC7 (ZD7) co-expression. (E) Immunoblot analysis of the WCL of sgZD7, sgZD12, or sgCtrl THP-1 cells differentiated with PMA, primed with LPS, and activated with Nig for 1 h. (F) ELISA of human IL-1β in the cell culture medium of THP-1 cells in (E). (G) Immunoblot analysis of NLRP3 and cleaved GSDMD (NT) in the WCL of WT or NLRP3-KO THP-1 cells that were reconstituted with the NLRP3 WT or CS mutant, differentiated with PMA, primed with LPS, and activated with Nig for 1 h. (H) ELISA of human IL-1β in the cell culture medium in (G). Data with error bars are mean ± SEM. *p < 0.05, **p < 0.01, ***p < 0.001, as determined by unpaired Student’s t test.

Thus far, our results strongly suggest that ZDHHC7-catalyzed NLRP3 Cys126 S-palmitoylation is important for NLRP3 inflammasome activation through regulating NLRP3 TGN/dTGN localization and ASC recruitment in macrophages. In contrast, recent work has reported that NLRP3 was palmitoylated on Cys841 by ZDHHC12 and that it terminated the inflammasome signaling through regulation of NLRP3 degradation.^53^ Therefore, we compared ZDHHC7-catalyzed Cys126 palmitoylation and ZDHHC12-catalyzed Cys841 modification. We deleted *ZDHHC7* or *ZDHHC12* in THP-1 cells to assess the NLRP3 *S*-palmitoylation. *ZDHHC7* deletion decreased the *S*-palmitoylation of NLRP3 in both the PMA-differentiated and inflammasome-activated THP-1 cells, whereas *ZDHHC12* deletion only decreased the NLRP3 *S*-palmitoylation in inflammasome-activated THP-1 cells (Figure 7A). This suggested that NLRP3 *S*-palmitoylation mediated by ZDHHC7 can occur regardless of the activation stage, while ZDHHC12-catalyzed NLRP3 *S*-palmitoylation was induced after NLRP3 activation.

Next, we sought to evaluate the difference between NLRP3 Cys126 and Cys841 (mouse NLRP3 number) palmitoylation. Through reconstituting NLRP3 WT and modification site mutants into the NLRP3-KO THP-1 cells, we found that all NLRP3 mutants, C130S, C844S, and C130/844S (human NLRP3 number) had decreased *S*-palmitoylation. C844S decreased about 40% of the modification level, while C130S decreased 80%, similar with the C130/844S mutant (80%) (Figure 7B). Given that palmitoylation of NLRP3 Cys844 terminated inflammasome signaling,^53^ whereas Cys130 palmitoylation was required for NLRP3 activation, we think the explanation for this result is that, in the absence of Cys130 palmitoylation, NLRP3 will not be activated, and Cys844 will not be modified, indicating a sequential palmitoylation process on Cys130 and Cys844. Additionally, we further determined NLRP3 *S*-palmitoylation by expressing mouse NLRP3 WT and mutants in HEK293T cells and found that NLRP3 C126A had a much lower modification level than C841A in the resting state (Figure 7C and 7D), and the C126/841A double mutant did not further decreased the modification level compared with C126A (Figure 7D), which is consistent with the results of human NLRP3 in THP-1 cells (Figure 7B). Moreover, NLRP3 *S*-palmitoylation was strongly promoted by ZDHHC7 co-expression (Figure 7D) but only marginally induced by ZDHHC12 (Figure 7C), further confirming that ZDHHC7-mediated Cys126 palmitoylation occurs in the resting state, while ZDHHC12-mediated Cys841 modification mainly occurs after NLRP3 activation.

Lastly, we aimed to compare the effects of NLRP3 *S*-palmitoylation between these two sites, catalyzed by two different ZDHHCs, on NLRP3 inflammasome regulation. Our data indicated that *ZDHHC7* deletion inhibited, while *ZDHHC12* KO enhanced, GSDMD cleavage and IL-1β secretion in activated THP-1 cells (Figures 7A, 7E, and 7F), which is consistent with the above results and a previous study.^53^ In THP-1 cells that were depleted of endogenous human NLRP3, the C130S or C130/844S mutant-reconstituted cells exhibited decreased GSDMD cleavage and IL-1β secretion compared with WT NLRP3-reconstituted cells, while C844S-reconstituted THP-1 showed enhanced GSDMD cleavage and IL-1β secretion (Figures 7G and 7H), suggesting that NLRP3 Cys126 (Cys130 for human NLRP3) palmitoylation promotes NLRP3 inflammasome activation, whereas palmitoylation of Cys841 (Cys844 for the human number) terminates inflammasome signaling in macrophages. These results indicate that NLRP3 inflammasome signaling is precisely and sequentially regulated by *S*-palmitoylation on different sites that are catalyzed by different ZDHHCs to tune the inflammasome process.

## DISCUSSION

In this study, we demonstrated that NLRP3 is palmitoylated on Cys126 by the palmitoyl-acyltransferase ZDHHC7. This palmitoylation event is important for NLRP3 localization at the TGN and dTGN as well as ASC recruitment and oligomerization and, thus, inflammasome activation. Cys126 is in the NLRP3 linker region between the Pyrin and NACHT domains^45^ and near the polybasic region that contributes to NLRP3 TGN localization.^23,24,49^ Our data showed that the NLRP3 *S*-palmitoylation site mutant disrupts its TGN/dTGN localization (Figure 4), and mutation of the polybasic region also abolishes NLRP3 Cys126 *S*-palmitoylation (Figures S8A–S8F). We think the likely explanation for this is that the polybasic region is required for transient NLRP3 interaction with TGN-localized ZDHHC7 (Figures S8G and S8H), and ZDHHC7-catalyzed palmitoylation then stabilizes NLRP3 Golgi apparatus localization. Therefore, it appears that both the polybasic residues and Cys126 *S*-palmitoylation contribute to localization of NLRP3 at the TGN.

Our data showed that NLRP3 Cys126 palmitoylation is important for ASC recruitment by activated NLRP3 and the consequential ASC oligomerization, which is likely a secondary effect of the TGN localization of resting NLRP3 and dTGN localization after activation. Suppressing NLRP3 Cys126 palmitoylation by depleting ZDHHC7, the major palmitoyl-acyltransferase of NLRP3 Cys126 palmitoylation, by pharmacologically inhibiting ZDHHC7 with inhibitors or by mutating the NLRP3 modification site Cys126, dramatically inhibited caspase-1/GSDMD cleavage and cytokine secretion in macrophages and in mouse models, supporting that ZDHHC7-mediated NLRP3 Cys126 palmitoylation is important for NLRP3 inflammasome activation. Our study shows that NLRP3 is palmitoylated in the resting state and that the palmitoylation is enhanced in LPS-primed BMDMs or PMA-differentiated THP-1 cells (Figure 1A and 1B). Previous work has suggested that LPS could significantly accelerate palmitic acid synthesis by inducing the expression of fatty acid synthase (FASN) and the maturation of sterol regulatory element binding protein 1c (SREBP-1c) in macrophages,^54,55^ which might contribute to the enhanced NLRP3 *S*-palmitoylation in primed macrophages. After activation with ATP or nigericin, NLRP3 *S*-palmitoylation remains (Figure 1A, 1B, and 1G), suggesting that *S*-palmitoylation may continue to stabilize NLRP3 on dTGN, which facilitates its transport to the MTOC and recruitment of adaptors to mediate inflammasome activation.

Recently, Wang and colleagues reported that NLRP3 is palmitoylated on Cys841 by ZDHHC12 and that this negatively regulates NLRP3 inflammasome signaling,^53^ which is distinct from our study. We compared ZDHHC7-mediated NLRP3 Cys126 palmitoylation and ZDHHC12-mediatd Cys841 palmitoylation and found that (1) NLRP3 Cy126 palmitoylation mediated by ZDHHC7 occurs even in the resting state, while ZDHHC12-catalyzed NLRP3 Cys841 palmitoylation is induced after NLRP3 activation; (2) NLRP3 Cys126 palmitoylation occurs more extensively than that of Cys841 palmitoylation in resting macrophages; and (3) NLRP3 Cys126 palmitoylation promotes NLRP3 inflammasome activation, while Cys841 palmitoylation terminates NLRP3 inflammasome signaling in macrophages. Therefore, NLRP3 inflammasome signaling is precisely and collaboratively regulated by the ZDHHC7-mediated NLRP3 Cys126 palmitoylation and ZDHHC12-promoted Cys841 palmitoylation in different stages of inflammasome signaling in a sequential process. Wang and colleagues also found that NLRP3 Cys841 palmitoylation promotes NLRP3 degradation through chaperone-mediated autophagy. However, in our study, the NLRP3 total protein level remained the same with 2-BP/MY-D4 incubation, *ZDHHC7*/*ZDHHC12* deletion, or NLRP3 Cys126/Cys841 (human Cys130/Cys844) mutation in macrophages (Figures S2, S3, and 7). One possible explanation for the difference is that we used a strong detergent (2.5% or 4.0% SDS) to solubilize all proteins, while Wang and co-workers used no detergent or 1% NP-40 lysis buffer, which may not be able to extract membrane-bound NLRP3.

Another very recent work from Zheng et al. further demonstrated that ZDHHC5-mediated NLRP3 *S*-palmitoylation on the Cys834/835 (mouse NLRP3 numbers), located at the NLRP3-NEK7 interface, could promote NLRP3-NEK7 association and enhance NLRP3 inflammasome assembly.^56^ Our study and the previous reports point to the following model. In the resting stage, NLRP3 is palmitoylated by ZDHHC7, which promotes NLRP3 TGN localization. After NLRP3 activation, Cys126 palmitoylation persists and ensures NLRP3 localization on the dTGN and its transport to the MTOC, where ZDHHC5-mediated Cys834/835 *S*-palmitoylation enhances NLRP3-NEK7 interaction. In other words, ZDHHC7-promoted Cys126 palmitoylation and ZDHHC5-promoted Cys834/835 palmitoylation both contribute to NLRP3 inflammasome activation via distinct mechanisms. In a later stage of inflammasome activation, ZDHHC12 is turned on to increase Cys841 palmitoylation and terminate inflammasome signaling. Therefore, NLRP3 *S*-palmitoylation on different sites have distinct functions, just like different NLRP3 phosphorylation sites mediated by distinct kinases in different stages of inflammasome process could have different regulatory functions.^57–61^ Such site-dependent regulatory roles of *S*-palmitoylation will likely be found in other proteins.

ZDHHC7-catalyzed NLRP3 *S*-palmitoylation is conserved in mouse and human macrophages, and the regulatory mechanism contributes to NLRP3 inflammasome activation *in vivo*. Therefore, small-molecule inhibitors of palmitoyl-acyltransferase ZDHHC7 or directly targeting NLRP3 Cys126 residue can potentially suppress inflammasome activation and thus may be useful for treating inflammation and autoimmune diseases. The observation that ZDHHC7 and ZDHHC12 have opposing and sequential regulatory effects on the NLRP3 inflammasome process also suggests that it is beneficial to have ZDHHC7-specific inhibitors to block the initiation of NLRP3 activation for treating inflammasome hyperactivation in human autoimmune diseases.

### Limitations of the study

In this study, we elucidated that NLRP3 is *S*-palmitoylated by ZDHHC7 on Cys126, which is important for NLRP3 TGN/dTGN localization and, consequently, inflammasome activation. This conclusion is extensively supported by the combined use of Cys126 mutation and the KO/inhibition of ZDHHC7 in macrophages and *in vivo*. However, one limitation is that we could not use mass spectrometry to identify the peptide containing Cys126, making it challenging to verify Cys126 palmitoylation by mass spectrometry. This is due to multiple reasons, including difficulty in getting a suitable amount of modified NLRP3 protein from macrophages, the labile nature of the modification,^62,63^ and the polybasic nature of the modification site, making it difficult to get proteolytic peptides suitable for mass spectrometry. The difficulty in obtaining NLRP3 protein also prevented us from carrying out *in vitro* palmitoylation assays. We note that, while NLRP3 protein has been recombinantly expressed and its structure was determined by cryoelectron microscopy (cryo-EM), the purified protein is locked in an inhibited conformation that cannot bind to NEK7 (unless they are co-expressed),^64^ implying that the purified NLRP3 was in an inactive state and could not be used for *in vitro* reconstitution. In addition, interaction of NLRP3 with ZDHHC7 may needthe TGN membrane to support their association, making it very tricky to perform the interaction assays *in vitro*. Therefore, more studies about NLRP3 palmitoylation and activation need to be carried out through cell-free *in vitro* assays in the future.

## STAR★METHODS

### RESOURCE AVAILABILITY

#### Lead contact

Further information and requests for resources and reagents should be directed to and will be fulfilled by the lead contact, Hening Lin (hl379@cornell.edu).

#### Materials availability

All unique and stable materials generated in this study are listed in the key resources table and available from the lead contact with a completed Materials Transfer Agreement.

#### Data and code availability

- Original imaging data for microscopy, gels and blots have been deposited at Mendeley Data (https://data.mendeley.com/datasets/7fr9nsybbs/2) and are publicly available as of the data of publication. The DOI of original data files is listed in the key resources table. This paper analyzes existing, publicly available data, accession number for the dataset is listed in the key resources table.
- This paper does not report the original code.
- Any additional information required to reanalyze the data reported in this paper is available from the lead contact upon request.

### EXPERIMENTAL MODEL AND STUDY PARTICIPANT DETAILS

#### Mice

B6.129P2(FVB)-Zdhhc7tm1.2Lusc/Mmmh (RRID: MMRRC_043511-MU) was ordered from the Mutant Mouse Resource and Research Center (MMRRC) at the University of Missouri.^26^ C57BL/6 mice were purchased from the Jackson Laboratories (Bar Harbor, ME). *Zdhhc3*, *Zdhhc6*, *Zdhhc9* knock-out mice with C57BL/6 background, and *Nlrp3*-C126A knock-in mice of C57BL/6 background were generated in this study through editing with CRISPR/Cas9 technology by STEM Cell and Transgenic Mouse Facility of Cornell University, the genes deletion or mutation were confirmed with real-time PCR and Sanger sequencing results, respectively. Mouse strains used and generated in this study are also listed in the key resources table. All the mice were kept in a specific-pathogen-free facility and provided with standard laboratory chow, housing in a barrier unit within individually ventilated cages in a room maintained with humidity of 65–75%, 12 h light/dark cycles, and at 22 ± 1°C. 8–10 weeks old mice, matched for age and sex (usually males and females mixed), were used for the animal models as described in the method details section below. All the animal experiments were approved by Cornell University’s Institutional Animal Care and Use Committee.

#### Cell culture studies

Human HEK293T cells were grown in Dulbecco’s modified Eagle’s medium (DMEM, Gibco) supplemented with 10% fetal bovine serum (FBS, Gibco), 100 units/mL Penicillin, 100 μg/mL Streptomycin, and 250 ng/mL Amphotericin B. Human monocytic THP-1 cells, THP-1 with NLRP3 knockout (NLRP3-KO THP-1 cells, ordered from InvivoGen, verified by immunoblot for NLRP3 knockout), THP-1 stable cell constructs with NLRP3 WT/mutants reconstitution, and THP-1 stable cell constructs with *ZDHHC7/12* deletion were cultured in Roswell Park Memorial Institute 1640 medium (RPMI 1640, Gibco) containing 10% heat-inactivated FBS, 100 units/mL Penicillin, 100 μg/mL Streptomycin, and 250 ng/mL Amphotericin B. Mouse bone marrow-derived macrophages (BMDMs) were differentiated from mouse bone marrow cells, and human peripheral blood mononuclear cells (PBMCs) were ordered from ATCC (PCS-800–011) and differentiated into macrophages following the procedures in the method details section below. All the cells were cultured in a 5% CO_2_ incubator at 37°C.

### METHOD DETAILS

#### Plasmids, antibodies, and reagents

pcDNA3-N-Flag-NLRP3 plasmid encoding mouse NLRP3 (Addgene plasmid # 75127) was a gift from Bruce Beutler.^65^ pcDNA3-Myc-ASC plasmid encoding human ASC (Addgene plasmid # 73952) and NLRP3-GFP plasmid (Addgene plasmid # 73955) were gifts from Christian Stehlik.^66,67^ Plasmid encoding human HA-ASC was obtained from GenScript. Flag-NLRP3 or NLRP3-GFP Cys to Ser (CS) mutants were constructed by site-directed mutagenesis. Plasmids encoding murine ZDHHC1–23 or the enzymatically inactive mutant ZDHHS7 were kind gifts from Dr. Masaki Fukata, National Institutes of Natural Sciences of Japan.

Immunoblot antibodies for NLRP3 (D4D8T), IL-1β (D4T2D), GSDMD (E7H9G), ASC/TMS1 (D2W8U), phospho-p65 (Ser536, 93H1), and HA-Tag (C29F4) were ordered from Cell Signaling Technology. Antibodies of Caspase-1 (Casper-1) and NLRP3 (Cryo-2) were purchased from AdipoGen. Antibodies for GSDMD (EPR19828) and ZDHHC7 were ordered from Abcam. Antibodies of β-actin (C4) for immunoblot and TGN38 (B-6) for immunofluorescence staining were purchased from Santa Cruz Biotechnology. Antibody for FLAG Tag (A8592) was ordered from Sigma.

2-Bromopalmitic acid (2-BP, CAS: 18263–25-7), lipopolysaccharide (LPS) of *Escherichia coli* O111:B4 or O127:B8, and Phorbol 12-myristate 13-acetate (PMA, CAS: 16561–29-8) were obtained from Sigma. Adenosine 5’-triphosphate disodium salt (ATP, CAS: 987–65-5), poly(dA:dT), and nigericin (Nig, CAS: 28643–80-3) were from InvivoGen. Cross linker disuccinimidyl suberate (DSS) was from ThermoFisher Scientific. Click chemistry reagent TAMRA-azide was from Lumiprobe, tris [(1-benzyl-1H-1,2,3-triazol-4-yl) methyl] amine (TBTA) were from TCI Chemicals, tris(2-carboxyethyl) phosphine HCl (TCEP hydrochloride) was from Millipore, and CuSO_4_ was from Mallinckrodt Chemical Works. Alkyne 14 (Alk14), 2-BP-Alk and MY-D4 were synthesized in our laboratory. For determination of cell culture media or serum cytokines, mouse IL-1β/IL-1F2 Quantikine ELISA kit, mouse IL-6 Quantikine ELISA kit, mouse IL-18/IL-1F4 ELISA kit, mouse TNF-alpha Quantikine ELISA Kit, mouse HMGB1/HMG-1 ELISA kit, and human IL-1β/IL-1F2 Quantikine ELISA kit were ordered from R&D. Control siRNA-A (sc-37007), siRNA targeting human *ZDHHC7* gene (sc-93249) and mouse *Zdhhc7* gene (sc-155507) were purchased from Santa Cruz Biotechnology.

#### Cell culture, transfection, and co-immunoprecipitation

ZDHHC7 knockout HEK293T was obtained as previously described,^26,27^ western blot and quantitative real-time PCR (Q-PCR) were used to confirm the knockout of ZDHHC7. For HEK293T cells transfection, polyethylenimine hydrochloride (PEI, Polysciences) was used as the transfection reagent. Briefly, HEK293T cells were seeded one day before transfection in 10-cm dishes with DMEM media containing 10% FBS. The next day plasmids encoding indicated proteins were diluted with Opti-MEM I Reduced Serum Medium (Thermofisher Scientific) and PEI was added at a ratio of 1:3 for DNA: PEI, then the DNA-PEI mixture was incubated at room temperature for 30 min and added into the HEK293T dishes in a drop-wise manner. After 36–48 h, cells were collected or treated for further analysis.

siRNA transfection in THP-1 cells or NLRP3-KO THP-1 cells was performed with transfection reagent Fugene SI according to manufacturer’s instruction. Briefly, THP-1 cells were seeded in 12-well plate and differentiated with 10 ng/mL PMA. The second day, cell media was changed with RPMI 1640 containing 5% heat inactivated FBS. 10 pmol RNAi duplex was diluted into 50 μL Opti-MEM I Reduced Serum Medium, 3 μL Fugene SI was diluted into another 50 μL Opti-MEM I Reduced Serum Medium, the diluted RNAi duplex and diluted Fugene SI were combined and incubated at room temperature for 15 min, before RNAi-Fugene mixture was added into the cell wells in a drop-wise manner. Cells were incubated for 36–48 h and treated for further analysis.

For co-immunoprecipitation (Co-IP) of Flag-NLRP3 with ASC expressed in HEK293T cells, after transfection cells were lysed with RIPA buffer (50 mM Tris-HCl, pH 7.4, 150 mM NaCl, 1% NP-40, 0.5% deoxylcholate sodium, 0.1% SDS) containing Protease Inhibitor Cocktail (Sigma, Cat. P8849) and then passed through a 21-G needle 20 times before centrifugation at 9000×g for 5 min. Flag-NLRP3 was immunoprecipitated using anti-FLAG M2 agarose beads (Sigma). Interacting proteins of Flag-NLRP3 were analyzed by immunoblotting analysis of the IP samples, cell lysates were analyzed to detect input proteins and loading controls. For Co-IP of endogenous NLRP3 with ZDHHC7 and ASC, mouse macrophage J774A.1, BMDMs and human macrophage THP-1 cells were lysed with RIPA buffer containing Protease Inhibitor Cocktail and nuclease (Pierce, Cat.88702) after LPS priming or with inflammasome activation, cell lysate was passed through a 21-G needle 20 times before centrifugation at 3000×g for 5 min, supernatant was incubated with anti-NLRP3 or anti-ZDHHC7 antibody as indicated and Protein A/G conjugated agarose beads (Santa Cruz) to immunoprecipitate endogenous NLRP3 or ZDHHC7 proteins, interacting proteins were accessed with immunoblotting analysis of the IP samples and cell lysate were determined to detect input samples and loading controls.

#### THP-1 stable cell line construction

To delete *ZDHHC7* or *ZDHHC12* in THP-1 cells, we constructed the THP-1 cells expressing Cas9 nuclease. Vector expressing Cas9 nuclease (GeneCopoeia) was transfected into HEK293T cells with packaging vector psPAX2 and envelope vector pMD2.G, 48 h later the cell culture media containing lentiviral particles was collected and filtered with 0.45 μm filters. The filtered media was then used for lentivirus transduction by infecting THP-1 cells with 8 μg/mL polybrene and incubated for 48 h before changing with fresh media and culturing for another 48 h. Infected THP-1 cells were selected by incubation with 400 ng/mL G-418 for 48 h, remaining live cells were grown up and validated for Cas9 expression. Next, we constructed the THP-1 cells with *ZDHHC7* and *ZDHHC12* KO using THP-1-Cas9 cells. Vectors expressing sgRNA targeting *ZDHHC7* or *ZDHHC12* (GeneCopoeia) were transfected into HEK293T cells along with psPAX2 and pMD2.G, filtered cell culture media containing lentiviral particles was used for transduction by infection THP-1-Cas9 cells, infected cells were selected by incubation with 1 μg/mL puromycin for 48 h, remaining live cells were grown up, validated for ZDHHC7 or ZDHHC12 expression by immunoblotting analysis and Q-PCR, and used for further assays. Sequences of sgRNA are listed in Table S2.

For the NLRP3-reconstituted THP-1 stable cell line construction, WT and mutated NLRP3 cDNA were constructed into pCDH backbone and transfected into HEK293T cells with psPAX2 and pMD2.G, filtered cell culture media containing lentiviral particles was used for transduction by infection NLRP3-KO THP-1 cells, infected cells were selected by incubation with 1 μg/mL puromycin for 48 h, remaining live cells were grown up, validated for NLRP3 expression and relevant sequences, and used for further assays.

#### Macrophage preparation and stimulation

Mouse bone-marrow-derived macrophages (BMDMs) were prepared as described previously.^68,69^ Briefly, femur and tibia from 8 to 10 weeks aged mice were used to isolate BM cells, then BM cells were cultured in 10-cm petri dishes with DMEM media containing 20% FBS, 20 ng/mL M-CSF, 100 units/mL Penicillin, 100 μg/mL Streptomycin, and 250 ng/mL Amphotericin B. After culturing for five days, attached cells were detached with EDTA-PBS digestion buffer (PBS containing 10 mM EDTA and 2% FBS) and seeded into culture plates for further assays.

Human peripheral blood mononuclear cells (PBMC)-derived primary macrophages were prepared as described previously.^68,70^ Briefly, PBMCs ordered from ATCC (PCS-800–011) were thawed and cultured with RPMI 1640 media containing 10% FBS, 2 mM L-glutamine, 50 ng/mL M-CSF, and 25 ng/mL IL-10 in 12-well plates. After culturing for seven days, floating cells were washed away, the attached cells were mature macrophages and used for further experiments.

For NLRP3 inflammasome activation, mouse BMDMs, J774A.1 cells, and human PBMC-derived macrophages were cultured in DMEM media (for BMDMs and J774A.1 cells) or RPMI 1640 media (for human PBMC-derived macrophages) containing 10% FBS and primed with 200 ng/mL LPS for 4 h before removing LPS. 5 mM ATP or 10 μM nigericin were added to the cells and incubated for 45 min or 1 h, respectively. THP-1 cells or NLRP3-KO THP-1 cells were cultured in RPMI 1640 media containing 10% FBS and differentiated with 10 ng/mL PMA for 24 h before PMA removal. Then 200 ng/mL LPS was incubated for 4 h before LPS removal, 10 μM nigericin was added to the cells and incubated for another 1 h. Macrophage whole cell lysate and cell culture media were collected for immunoblotting analysis and ELISA assay. For 2-BP treatment, 5 μM or 10 μM 2-BP was used after LPS priming and removing, and together with ATP or nigericin incubation (J774A.1 cells, BMDMs and PBMC-derived macrophages), or 1 h before nigericin incubation (THP-1 and NLRP3-KO THP-1 cells).

For non-canonical inflammasome activation, mouse BMDMs were seeded in 12-well plate and primed with 200 ng/mL LPS for 4 h before removing LPS, then cells were transfected with 1 μg LPS using Lipofectamine 3000 Transfection Reagent (Thermofisher Scientific) and incubated for 6 h. For AIM2 inflammasome activation, LPS-primed mouse BMDMs were transfected with 2 μg poly(dA:dT) (InvivoGen) using Lipofectamine 3000 and incubated for 6 h.

Macrophages whole cell lysate was obtained by boiling with 2.5% SDS loading buffer after removing cell culture media and washing with PBS. This procedure was used to make sure that we extract the total proteins in cells, even those that were in aggregates and might be insoluble in other detergents.

#### ASC oligomerization assay

ASC oligomerization determination was modified from a previous protocol.^71^ After stimulation with ATP or nigericin for 30 min, macrophages were washed with cold PBS and then scraped off the plate and resuspended in 1% NP-40 of HEPES buffer. The cell suspension was passed through a 21-G needle 20 times and centrifuged at 4°C, 200 × g for 5 min. The supernatant was transferred into a new tube and centrifuged at 4°C, 5000 × g for 10 min. The new supernatant was kept for immunoblotting as loading control and the pellet was washed with PBS twice and cross-linked with 2 mM DSS for 30 min at room temperature. After centrifuging at 4°C, 5000 × g for 10 min, the pellet was resuspended with 30 μL SDS-PAGE protein loading buffer and determined by immunoblotting analysis.

#### Click chemistry to detect S-palmitoylation

Click chemistry was used for *S*-palmitoylation determination in this study, following the protocol as previously described.^26,27^ After plasmids transfection and 50 μM Alk14 incubation for 6 h in HEK293T cells, Flag-NLRP3 was immunoprecipitated with anti-Flag M2 resin, NLRP3-GFP was immunoprecipitated with GFP-Trap (Chromotek). The agarose beads were washed with IP washing buffer (25 mM Tris-HCl, pH 8.0, 150 mM NaCl, 0.2% NP-40) three times and resuspended with 20 μL of IP washing buffer. To this bead suspension, click chemistry reagents (2 mM TAMRA-azide, 10 mM TBTA, 40 mM CuSO_4_, 40 mM TCEP, 1 μL each) were added and incubated at room temperature avoiding light. After 30 min, the reactions were stopped by adding 10 μL SDS loading buffer and boiled at 95°C for 10 min. For hydroxylamine treatment, the boiled samples were centrifuged, and supernatants were transferred to a new tube. Hydroxylamine was added to the supernatants to be final concentration of 0.5 M and the samples were boiled for another 5 min. Alk14 label signals were assessed by SDS-PAGE and in-gel fluorescence analysis with ChemiDoc Imagers (Bio-Rad).

For Alk14 labeling of NLRP3 in BMDMs and THP-1 cells, after LPS priming, inflammasome activation and Alk14 incubation, 1×10^7^ BMDMs were lysed with 0.6 mL 4% SDS buffer (4% SDS, 150 mM NaCl, 50 mM TEA, pH7.4) containing protease inhibitors and nuclease (Pierce), to make sure that all the NLRP3 protein (including membrane-bound NLRP3) was obtained after cell lysis. The protein concentration was measured by BCA Protein Assay Kit (Pierce) and normalized. Click chemistry reagents (5 mM Biotin-azide, 2 mM TBTA, 50 mM CuSO_4_, 50 mM TCEP, 30 μL for each) were added and incubated at 37°C avoiding light. After 2 h, the reaction was stopped, and protein was precipitated by methanol and chloroform. After centrifugation at 4°C and 4000 × g for 30 min, the clear upper phase was removed, and methanol was added to wash the central protein phase. Then the protein pellet was collected with another centrifugation at 4°C and 4000 × g for 30 min. The protein pellet was resuspended with 200 μL of 2% SDS buffer (2% SDS and 50 μM EDTA in PBS) and diluted with PBS to 2 mL. Streptavidin agarose beads were added to the sample and incubated at room temperature for 1 h. The beads were collected and washed with IP washing buffer three times. The washed beads were suspended in 30 μL SDS loading buffer and boiled at 95°C for 10 min. The proteins pull-downed by the streptavidin beads, which were labeled with Alk14, were assessed by immunoblotting analysis with anti-NLRP3 antibody. PBS-diluted protein resuspension without streptavidin beads incubation was accessed with immunoblot to detect the input samples and loading controls.

#### Acyl-biotin exchange to detect S-palmitoylation

The acyl-biotin exchange (ABE) procedure was modified as previously described.^26,72^ To determine the palmitoylation of total NLRP3 protein (including membrane-bound NLRP3), cells were lysed with ABE lysis buffer (100 mM Tris-HCl pH 7.2, 150 mM NaCl, 2.5% SDS, 10 mM *N*-ethylmaleimide (NEM), protease inhibitors, nuclease) to block the free cysteines of total proteins with NEM. After quenching with 100 mM 2,3-dimethyl 1,3-butadiene and precipitating with one-tenth volume of chloroform, supernatant was incubated with 0.5 M hydroxylamine (HAM+) or NaCl (HAM-). All samples were incubated with Biotin-HPDP and proteins were precipitated with one-volume of chloroform, four volumes of methanol and three volumes of ddH_2_O. Protein pellet was resuspended with ABE Resuspension Buffer (100 mM Tris-HCl pH 7.2, 150 mM NaCl, 5 mM EDTA, 2.5% SDS, 8 M urea) after washing with four volumes of methanol. Then the resuspended protein was diluted with PBS and palmitoylated proteins (Biotin-HPDP-linked) were immunoprecipitated with streptavidin pull-down. IP and input samples were used for SDS-PAGE and immunoblot, palmitoylated NLRP3 was detected with anti-NLRP3 in the IP samples.

#### Real-time quantitative PCR

Total RNA was extracted by E.Z.N.A. Total RNA Kit I (Omega Bio-tek) according to manufacturer’s instruction. cDNA was synthesized with SuperScript VILO cDNA Synthesis Kit (Invitrogen). Real-time quantitative PCR (Q-PCR) was performed in triplicate using 2 × Universal SYBR Green Fast qPCR Mix (ABclonal) by QuantStudio 7 Flex Real-Time PCR System. Relative expression of genes was calculated using a standard curve method and normalized to the *β-actin (Actb)*. Primers for Q-PCR are listed in Table S1.

#### Immunofluorescence imaging

After plasmids transfection or stimuli treatment, cells were washed with cold PBS and fixed with 4% paraformaldehyde solution (4% PFA, Santa Cruz) at 4°C for 15 min, and permeabilized with 0.2% Triton X-100 in PBS at room temperature for 10 min. The cells were blocked with 3% bovine serum albumin (BSA) in PBST buffer (PBS containing 0.5% Tween 20) at room temperature for 1 h. Then specific primary antibodies were added at a ratio of 1:200 dilution in 3% BSA-PBST buffer and incubated at 4°C overnight. The cells were washed with PBS three times, and fluorescently conjugated secondary antibodies were used at a ratio of 1:1000 dilution in 3% BSA-PBST buffer and incubated at room temperature for 1 h in dark before PBS washing for another three times. NLRP3-Flag was stained with anti-Flag antibody conjugated with Alexa Fluor 488 at a ratio of 1: 500 dilutions in 3% BSA-PBST buffer and incubated at 4°C overnight. DAPI Fluoromount-G Mounting Medium (Southern Biotech) was used for nucleus staining. The stained cells were photographed with LSM710 Confocal Microscope (Zeiss). Quantification of NLRP3 colocalization with TGN markers, TGN38 or 58K Golgi, was analyzed by ImageJ with the method of Pearson’s correlation coefficient (PCC),^73^ 4–6 fields of vision from multiple slides were used for analysis in each group.

#### Endotoxic shock mouse model

The procedure of LPS-induced endotoxic shock model was modified as previously^71^. Wildtype and *Zdhhc7* knockout mice with B6.129P2(FVB) background, or Wildtype and NLRP3-C126A knock-in mice with C57BL/6 background, were injected intraperitoneally (i.p.) with 35 mg/kg of body weight LPS (lipopolysaccharides from *Escherichia coli* O111:B4, obtained from Sigma, catalog number: L2630, dissolved in sterile PBS). Eight hours later, the mice were sacrificed, and serum was collected. For 2-BP administration, wildtype mice were administered with DMSO (vehicle control, 2 mL/kg), or 25 mg/kg 2-BP by i.p. injection. After 12 h, 35 mg/kg of LPS was injected by i.p. Eight hours later, mice were sacrificed, and the blood was collected via cardiac puncture to prepare serum. IL-1β, IL-6, IL-18, TNF-α, and HMGB-1 concentrations in mouse serum were detected as indicated by ELISA Kits (R&D) following the manufacturer’s instruction. Peritoneal exudate cells (PEC) of mouse with LPS-induced endotoxic shock were isolated and NLRP3 palmitoylation was determined by acyl-biotin exchange (ABE) assay.

#### Mono sodium urate crystal (MSU)-induced peritonitis

The procedure of MSU-induced peritonitis model was modified as previously^71^. Wildtype or *Zdhhc7* knockout mice with B6.129P2(FVB) background were injected intraperitoneally (i.p.) with 1 mg/kg of body weight LPS (Sigma, L2630, in 200 μL sterile PBS) for 3 h and then with 200 μL MSU suspension (i.p., 10 mg/mL, in sterile PBS) for each mouse using a 1-mL syringe, 6 h after MSU administration the mice were sacrificed, blood was collected via cardiac puncture to prepare serum. Then, inject 3 mL PBS i.p. into each mouse using a 5-mL syringe and collect abdominal contents by peritoneal lavage. Cell numbers of peritoneal exudate cells (PEC) were counted with Bio-Rad TC20 Automated Cell Counter, infiltrated neutrophils and macrophages in peritoneal lavage fluids were determined with flow cytometry by staining and gating with CD45^+^CD11b^+^Gr-1^+^ (neutrophils) or CD45^+^CD11b^+^F4/80^+^ (macrophages) and analyzed with Thermo Fisher Attune NxT Analyzer.

### QUANTIFICATION AND STATISTICAL ANALYSIS

Data are shown as mean ± SEM, all presented data are representative results of at least three independent repetition, unless specifically indicated. Statistical analysis was performed with GraphPad Prism 9 (Graph-Pad Software), statistics were analyzed using two-tailed Student’s t-test, Log rank (mantel-cox) test, one-way or two-way ANOVA as described in the figure legends. Differences of data were considered to be significant at p ≤ 0.05 and are indicated by *, those at p ≤ 0.01 are indicated by **, and those at p ≤ 0.001 are indicated by ***.

## Supplementary Material

1

## Figures and Tables

**Table:** KEY RESOURCES TABLE

REAGENT or RESOURCE	SOURCE	IDENTIFIER

Antibodies		

Anti-mouse IgG, HRP-linked	Cell Signaling Technology	Cat# 7076; RRID:AB_330924
Anti-rabbit IgG, HRP-linked	Cell Signaling Technology	Cat# 7074; RRID:AB_2099233
Anti-NLRP3	Cell Signaling Technology	Cat# 15101; RRID:AB_2722591
Anti-ASC	Cell Signaling Technology	Cat# 67824; RRID:AB_2799736
Anti-HA-tag	Cell Signaling Technology	Cat# 3724; RRID:AB_1549585
Anti-IL-1β	Cell Signaling Technology	Cat# 12426; RRID:AB_2797907
Anti-Cleaved Gasdermin D (Asp275)	Cell Signaling Technology	Cat #36425; RRID:AB_2799099
Anti-phospho-p65 (Ser536)	Cell Signaling Technology	Cat# 3033; RRID:AB_331284
Anti-Gasdermin D	Cell Signaling Technology	Cat# 39754; RRID:AB_2916333
Anti-Caspase-1 (p20)	Adipogen Life Sciences	Cat# AG-20B-0042-C100; RRID:AB_2755041
Anti-NLRP3/NALP3	Adipogen Life Sciences	Cat# AG-20B-0014; RRID:AB_2490202
Anti-β-actin, HRP-linked	Santa Cruz Biotechnology	Cat# sc-47778 HRP; RRID:AB_626632
Anti-HA-probe, HRP-linked	Santa Cruz Biotechnology	Cat# sc-7392 HRP; RRID:AB_627809
Anti-TGN38	Santa Cruz Biotechnology	Cat# sc-166594; RRID:AB_2287347
Anti-TGN38	Novus Biologicals	Cat# NBP1–03495SS; RRID:AB_1522533
Anti-58K Glogi	Novus Biologicals	Cat# NB600–412SS; RRID:AB_2263623
Anti-Gasdermin D	Abcam	Cat# ab209845; RRID:AB 2783550
Anti-ZDHHC7	Abcam	Cat# ab138210
Anti-ZDHHC6 (C-TERM)	Sigma	Cat# SAB1304457
Anti-Flag-Tag,HRP-linked	Sigma	Cat#A8592; RRID:AB_439702

Biological samples		

Human Peripheral Blood Mononuclear Cells (PBMCs)	ATCC	ATCC# PCS-800–011
Mouse serum	B6.129P2(FVB)-*Zdhhc*7^+/+^ and *Zdhhc*7^−/−^ mice; C57Bl/6 mice; NLRP3-C126A mice.	N/A
Mouse peritoneal washings effusions	B6.129P2(FVB)-*Zdhhc*7^+/+^ and *Zdhhc*7^−/−^ mice.	N/A

Chemicals, peptides, and recombinant proteins		

2-Bromohexadecanoic Acid (2-bromopalmitate, 2-BP)	Sigma-Aldrich	Cat# 21604–1G; CAS:18263–25-7
Hydroxylamine Solution	Sigma-Aldrich	Cat# 438227–50ML; CAS 7803–49-8
Lipopolysaccharides (LPS) from *Escherichia coli* O111:B4	Sigma-Aldrich	Cat# L2630
Lipopolysaccharides (LPS) from *Escherichia coli* O127:B8	Sigma-Aldrich	Cat# L3129
Phorbol 12-myristate 13-acetate (PMA)	Sigma-Aldrich	Cat# P1585; CAS: 16561–29-8
Tris (2-carboxyethyl) phosphine HCl (TCEP hydrochloride)	Sigma-Aldrich	Cat# 75259; CAS: 51805–45-9
Alkyne-tagged Palmitic Acid Analog (Alkyne 14, Alk14)	This study	N/A
Alkyne-tagged 2-bromopalmitate (2-BP-Alk14)	This study	N/A
Adenosine 5’-triphosphate disodium salt (ATP)	InvivoGen	Cat# tlrl-atpl; CAS: 987–65-5
poly(dA:dT)	InvivoGen	Cat# tlrl-patn; CAS: 86828–69-5
Nigericin sodium salt	InvivoGen	Cat# tlrl-nig; CAS: 28643–80-3
TAMRA-azide	Lumiprobe	Cat# C7130; CAS: 825651–66-9
Tris [(1-benzyl-1H-1,2,3-triazol-4-yl) methyl] amine (TBTA)	TCI Chemicals	Cat# T2993; CAS: 510758–28-8
Control siRNA-A	Santa Cruz Biotechonology	Cat# sc-37007
siRNA Targeting Human *ZDHHC7* Gene	Santa Cruz Biotechonology	Cat# sc-93249
DSS (disuccinimidyl suberate)	Thermofisher Scientific	Cat# 21555; CAS: 68528–80-3
RPMI 1640 Medium	Thermofisher Scientific	Cat# 11875119
DMEM, high glucose	Thermofisher Scientific	Cat# 11965126
Fetal Bovine Serum, certified, United States	Thermofisher Scientific	Cat# 16000069
Antibiotic-Antimycotic (100X)	Thermofisher Scientific	Cat# 15240062
GlutaMAX^™^ Supplement	Thermofisher Scientific	Cat# 35050061
Opti-MEM^™^ I Reduced Serum Medium	Thermofisher Scientific	Cat# 31985070
Recombinant Mouse M-CSF (carrier-free)	Biolegend	Cat# 576406
Polyethylenimine Hydrochloride (PEI)	Polysciences	Cat# 24885–2
Fugene SI	Fugene	Cat# SI-1000
CUPRIC SULFATE, 5-HYDRATE, FINE CRYSTAL, AR (ACS)	Mallinckrodt Chemical Works	Cat# 4844
MY-D4	This study (Hong et al.)^37^ (Jiang et al.)^38^	N/A

Critical commercial assays		

CytoTox 96^®^ Non-Radioactive Cytotoxicity Assay	Promega	Cat# G1780
Mouse IL-1 beta/IL-1F2 Quantikine ELISA Kit	R&D SYSTEMS	Cat# MLB00C
Mouse IL-18/IL-1F4 ELISA	R&D SYSTEMS	Cat# 7625
Mouse TNF-alpha Quantikine ELISA Kit	R&D SYSTEMS	Cat# MTA00B
Mouse IL-6 Quantikine ELISA Kit	R&D SYSTEMS	Cat# M6000B
Human IL-1 beta/IL-1F2 Quantikine ELISA Kit	R&D SYSTEMS	Cat# DLB50
E.Z.N.A.^®^ Total RNA Kit I	Omega BIO-TEK	Cat# R6834–02
Applied Biosystems^™^ High-Capacity cDNA Reverse Transcription Kit	Fisher Scientific	Cat# 43–688-13
2X Universal SYBR Green Fast qPCR Mix	Abclonal	Cat# RK21203
ANTI-FLAG^®^ M2 Affinity Gel	Sigma-Aldrich	Cat# A2220
GFP-Trap^®^ Agarose	ChromoTek	Cat# gta-20
High-Capacity Streptavidin Agarose	Pierce	Cat# 20361
Protein A/G conjugated agarose beads	Santa Cruz Biotechonology	Cat# sc-2003

Deposited data		

Original western data for figures	This study; Mendeley Data	https://data.mendeley.com/datasets/7fr9nsybbs/2. (DOI:10.17632/7fr9nsybbs.2)
Expression of *ZDHHC* genes in human classical monocytes	Database of DICE (Schmiedel et al.)^36^	https://dice-database.org.

Experimental models: cell lines		

HEK293T	ATCC	ATCC# CRL-11268
DHHC7-KO HEK293T	Zhang et al.^26^	N/A
THP-1	ATCC	ATCC# TIB-202
NLRP3-KO THP-1	InvivoGen	Cat# thp-konlrp3z
J774A.1	ATCC	ATCC# TIB-67
NLRP3-KO THP1 with mouse NLRP3-WT	This study	N/A
sgRNA Scramble THP-1	This study	N/A
sgZDHHC7 THP-1	This study	N/A
sgZDHHC12 THP-1	This study	N/A
NLRP3-KO THP1 with mouse NLRP3-C126S	This study	N/A
NLRP3-KO THP1 with human NLRP3-WT	This study	N/A
NLRP3-KO THP1 with human NLRP3-C130S	This study	N/A
NLRP3-KO THP1 with human NLRP3-C130/844S	This study	N/A
NLRP3-KO THP1 with human NLRP3-C844S	This study	N/A

Experimental models: Organisms/strains		

Mouse: *Zdhhc7*^−/−^: B6.129P2(FVB)- *Zdhhc7*^*tm1.2Lusc*^/Mmmh	Mutant Mouse Resource and Research Center (MMRRC)	RRID: MMRRC_043511-MU
Mouse: *Zdhhc7*^+/+^: B6.129P2(FVB) (for control group)	Mutant Mouse Resource and Research Center (MMRRC)	N/A
Mouse: *Zdhhc3*^−/−^	STEM Cell and Transgenic Mouse Facility of Cornell University, this study.	N/A
Mouse: *Zdhhc6*^−/−^	STEM Cell and Transgenic Mouse Facility of Cornell University, this study.	N/A
Mouse: *Zdhhc9*^−/−^	STEM Cell and Transgenic Mouse Facility of Cornell University, this study.	N/A
Mouse: *NLRP3 C126A*	STEM Cell and Transgenic Mouse Facility of Cornell University, this study.	N/A

Oligonucleotides		

QPCR primers	This paper	Table S1
sgRNA sequences	This paper	Table S2

Recombinant DNA		

Flag-NLRP3	Shi et al.^65^	pcDNA3-N-Flag-NLRP3, Addgene plasmid # 75127.
pCDH vector	System Biosciences	pCDH-CMV-MCS-EF1-Puro, Addgene plasmid # 2082.
psPAX2 vector	Trono Lab Packaging and Envelope Plasmids (unpublished)	psPAX2, Addgene plasmid # 12260.
pMD2.G vector	Trono Lab Packaging and Envelope Plasmids (unpublished)	pMD2.G, Addgene plasmid # 12259.
Myc-ASC	Bryan et al.^66^	pcDNA3-Myc-ASC, Addgene plasmid #73952.
NLRP3-GFP	Khare et al.^67^	pEGFP-C2-NLRP3, Addgene plasmid # 73955.
pcDNA3.1(+)-C-HA-ASC	GenScript	Clone ID: OHu19678C
Flag-NLRP3-CS Constructs	This paper	N/A
NLRP3-GFP-CS Constructs	This paper	N/A
pCDH-CMV-MCS-EF1-Puro-Flag-NLRP3 and C126S Constructs	This paper	N/A
ZDHHCs (DHHC1–23)	Dr. Masaki Fukata, National Institutes of Natural Sciences of Japan; Zhang et al.	N/A
pCRISPR-LvSG03-ZDHHC7 sgRNA	GeneCopoeia	Plasmid #HCP257256-LvSG03–3-B
pCRISPR-LvSG03-ZDHHC12 sgRNA	GeneCopoeia	Plasmid #HCP314389-LvSG03–3-B
pLvC9NU-Cas9	GeneCopoeia	Plasmid #CP-LvC9NU-01
pCRISPR-LvSG03-Scrambled sgRNA	GeneCopoeia	Plasmid #CCPCTR01-LvSG03-B
pCDH-CMV-MCS-EF1-Puro-human NLRP3 and CS Constructs	This study	N/A

Software and algorithms		

Image Lab 6.1	Bio-Rad Laboratories, Inc.	https://www.bio-rad.com/en-us/product/image-lab-software?ID=KRE6P5E8Z#fragment-3
GraphPad Prism Version 9.5.0 (730)	GraphPad software, inc.	https://www.graphpad.com/
BioRender	BioRender	https://www.biorender.com
ImageJ 1.53k	National Institutes of Health	https://imagej.nih.gov/ij
Zotero 6.0.23	Zotero, Corporation for Digital Scholarship.	https://www.zotero.org/
FlowJo software Version 10.7	FlowJo LLC; BD	https://www.flowjo.com
ZEN (blue edition)	Carl Zeiss Microscopy GmbH	https://www.zeiss.com/corporate/us/home.html

## Highlights

- NLRP3 Cys126 is palmitoylated by ZDHHC7 in macrophages
- ZDHHC7-mediated NLRP3 Cys126 palmitoylation is critical for NLRP3 TGN localization
- Cys126 palmitoylation is vital for recruiting ASC and inflammasome assembly
- Cys126 palmitoylation promotes inflammasome activation in macrophages and *in vivo*
