# Supplementary material for: NLRP3 Cys126 palmitoylation by ZDHHC7 promotes inflammasome activation
Source: Cell Rep. Author manuscript; Available in PMC 2024 May 28. (PMC11130711; doi:10.1016/j.celrep.2024.114070)
Supplement: 1 [file NIHMS1988712-supplement-1.pdf]

Cell Reports, Volume *43*

Supplemental information

NLRP3 Cys126 palmitoylation by ZDHHC7 promotes inflammasome activation

Tao Yu, Dan Hou, Jiaqi Zhao, Xuan Lu, Wendy K. Greentree, Qian Zhao, Min Yang, Don-Gerard Conde, Maurine E. Linder, and Hening Lin

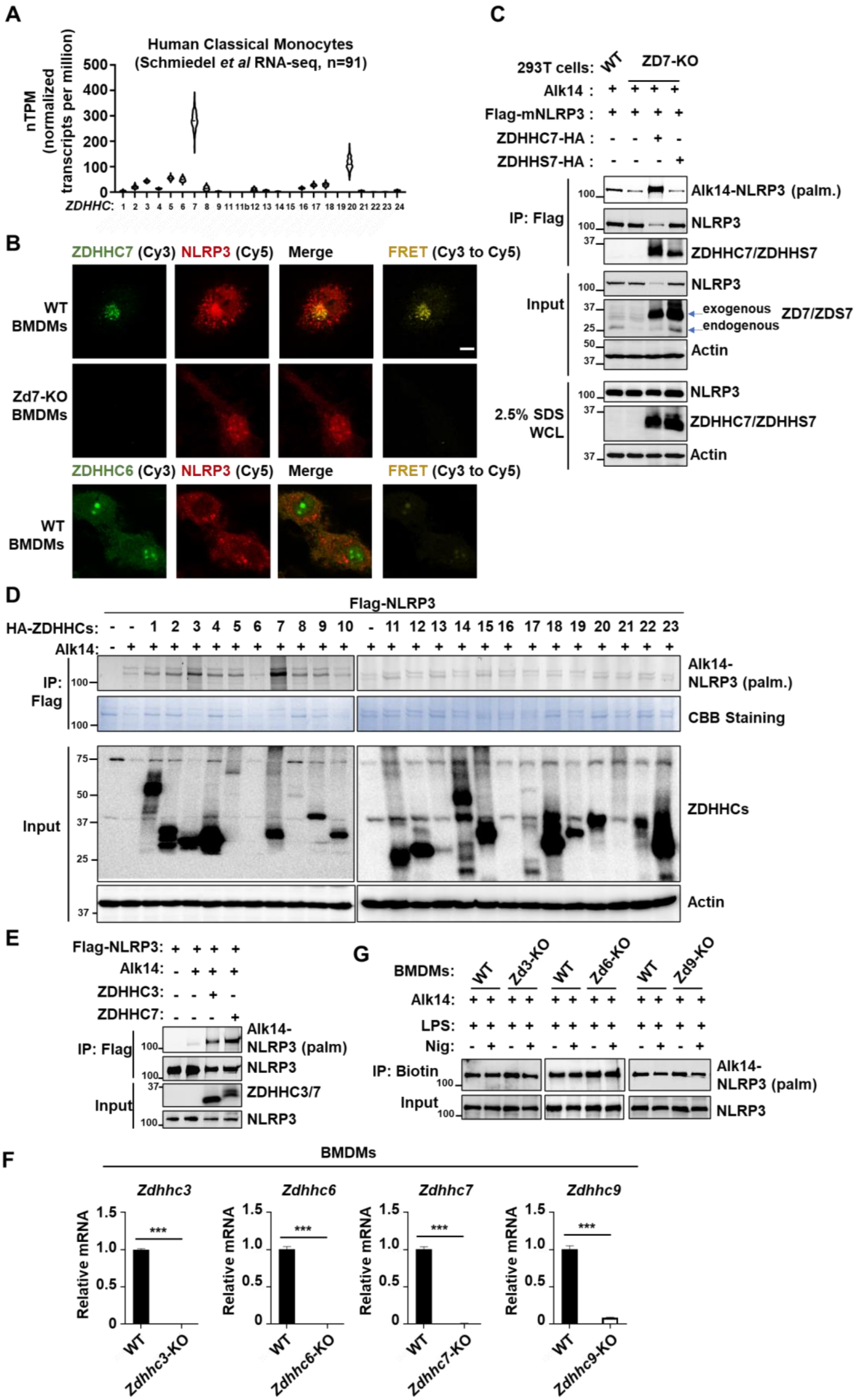

A
Human Classical Monocytes
(Schmiedel et al RNA-seq, n=91)
nTPM (normalized transcripts per million)
ZDHHC:
B
ZDHHC7 (Cy3) NLRP3 (Cy5) Merge FRET (Cy3 to Cy5)
WT BMDMs
Zd7-KO BMDMs
ZDHHC6 (Cy3) NLRP3 (Cy5) Merge FRET (Cy3 to Cy5)
WT BMDMs
C
293T cells: WT ZD7-KO
Alk14 :
Flag-mNLRP3 :
ZDHHC7-HA :
ZDHHS7-HA :
IP: Flag
Alk14-NLRP3 (palm.)
NLRP3
ZDHHC7/ZDHHS7
Input
NLRP3
exogenous
endogenous
ZD7/ZDS7
Actin
2.5% SDS WCL
NLRP3
ZDHHC7/ZDHHS7
Actin
D
Flag-NLRP3
HA-ZDHHCs:
Alk14:
IP: Flag
Alk14-NLRP3 (palm.)
CBB Staining
Input
ZDHHCs
Actin
E
Flag-NLRP3:
Alk14:
ZDHHC3:
ZDHHC7:
IP: Flag
Alk14-NLRP3 (palm)
NLRP3
Input
ZDHHC3/7
NLRP3
G
BMDMs: WT Zd3-KO WT Zd6-KO WT Zd9-KO
Alk14:
LPS:
Nig:
IP: Biotin
Alk14-NLRP3 (palm)
Input
NLRP3
F
BMDMs
Zdhhc3
Zdhhc6
Zdhhc7
Zdhhc9
Relative mRNA
WT
Zdhhc3-KO
Zdhhc6-KO
Zdhhc7-KO
Zdhhc9-KO

Figure S1. NLRP3 is palmitoylated by ZDHHC7 in macrophages, related to Figure 1.

(A) Expression of *ZDHHC* genes in human classical monocytes ($CD14^{high}$ $CD16^{-}$) generated from the database of Human Protein Atlas (https://www.proteinatlas.org) and DICE (https://dice-database.org, n=91)[S1]. **(B)** ZDHHC7 was stained with Cyanine 3 (Cy3) as a fluorescence resonance energy transfer (FRET) donor and NLRP3 was stained with Cyanine 5 (Cy5) as a FRET acceptor in LPS-primed BMDMs. The interaction between NLRP3 and ZDHHC7 in wildtype BMDMs was determined by the FRET signal, ZDHHC7 knockout (Zd7-KO) BMDMs and ZDHHC6 staining were used as negative controls. Scale bar: 5 μm. **(C)** Palmitoylation of Flag-NLRP3 expressed in wildtype (WT) HEK 293T cells, or ZDHHC7-deleted (ZD7-KO) HEK 293T cells reconstituted with wildtype ZDHHC7 or its enzymatic DHHC motif mutant (ZDHHS7). Palmitoylation of NLRP3 was detected using Alk14 labeling and in-gel fluorescence. **(D)** NLRP3 palmitoylation determination in HEK293T cells that were transfected with Flag-NLRP3 and each of the palmitoyl-transferases ZDHHCs (ZDHHC1-23) and incubated with 50 μM Alk14 probe, assessed by in-gel fluorescence and immunoblotting analysis. NLRP3 was tagged with Flag (Flag-NLRP3) and ZDHHCs were tagged with HA (HA-DHHCs). **(E)** NLRP3 palmitoylation determination in HEK293T cells that were transfected with Flag-NLRP3, ZDHHC3-HA, or ZDHHC7-HA as indicated. **(F)** Relative mRNA of *Zdhhc3, Zdhhc6, Zdhhc7*, and *Zdhhc9* in BMDMs determined by Q-PCR, showing the relevant *Zdhhc* was knocked-out successfully. Relative mRNA was normalized to *β-actin*. **(G)** Palmitoylation of NLRP3 in wildtype (WT) and *Zdhhc3*, *Zdhhc6*, *Zdhhc9*-deleted (Zd3-KO, Zd6-KO, Zd9-KO, respectively) BMDMs by Alk14 labeling and click chemistry assay. BMDMs were incubated with Alk14, LPS and nigericin (Nig) as indicated, cells were then lysed and proteins were conjugated with biotin-azide, labeled proteins were pulled down with streptavidin and blotted for NLRP3. Data with error bars represents mean ± SEM. $*p < 0.05$, $**p < 0.01$, $***p < 0.001$ as determined by unpaired Student's t test.

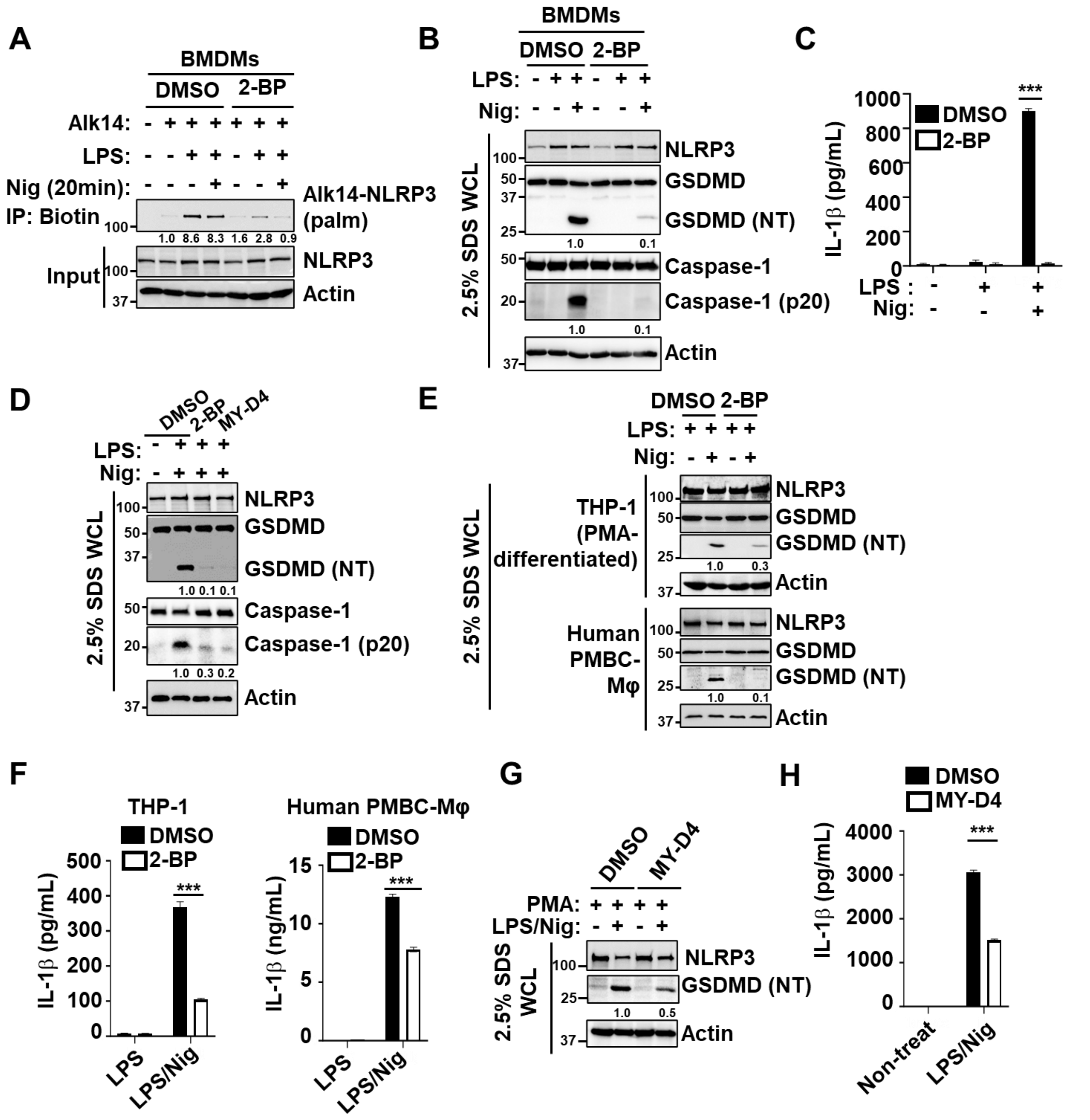

Figure S2. *S*-palmitoylation promotes NLRP3 inflammasome activation, related to Figure 1.

(A) *S*-Palmitoylation of NLRP3 is inhibited by 2-bromopalmitate (2-BP) in BMDMs. BMDMs were pre-treated with 2-BP for 1 h before Alk14 incubation, and LPS and nigericin were added as indicated. NLRP3 palmitoylation level was quantified and normalized to NLRP3 protein level in input samples. **(B)** Immunoblot analysis of NLRP3, GSDMD, Caspase-1, cleaved Caspase-1 (p20), and cleaved GSDMD N-terminal domain (NT) in whole cell lysate (WCL) of BMDMs treated with DMSO or 10 μM 2-BP and activated with 10 μM nigericin. Cells were lysed with lysis buffer containing 2.5% SDS to get total proteins. **(C)** ELISA determination of IL-1β in cell culture media of LPS-primed BMDMs in (**B**). **(D)** Immunoblot analysis of NLRP3, GSDMD, Caspase-1, cleaved Caspase-1 (p20), and cleaved GSDMD N-terminal domain (NT) in whole cell lysate of BMDMs

treated with DMSO, 10 μM 2-BP, or 20 μM MY-D4, and activated with 10 μM nigericin. Cells were lysed with lysis buffer containing 2.5% SDS to get total proteins. **(E)** Immunoblot analysis of NLRP3, GSDMD, and cleaved GSDMD N-terminal domain (NT) in whole cell lysate of phorbol 12-myristate 13-acetate (PMA)-differentiated THP-1 (top) and human peripheral blood mononuclear cells (PBMC)-derived primary macrophages (bottom) that were pre-treated with DMSO or 10 μM 2-BP for 1 h before activation with LPS and nigericin. **(F)** IL-1β determined by ELISA in cell culture media of THP-1 (left) and human PBMC-derived macrophages (right) in (**E**). **(G-H)** Immunoblot analysis of NLRP3 and cleaved GSDMD (NT) in whole cell lysate (**G**) and IL-1β determined by ELISA in cell culture media (**H**) of PMA-differentiated THP-1 cells pre-treated with DMSO or 20 μM MY-D4 for 1 h before activation with LPS and nigericin. Data with error bars are mean ± SEM. $*p < 0.05$, $**p < 0.01$, $***p < 0.001$ as determined by unpaired Student's t test.

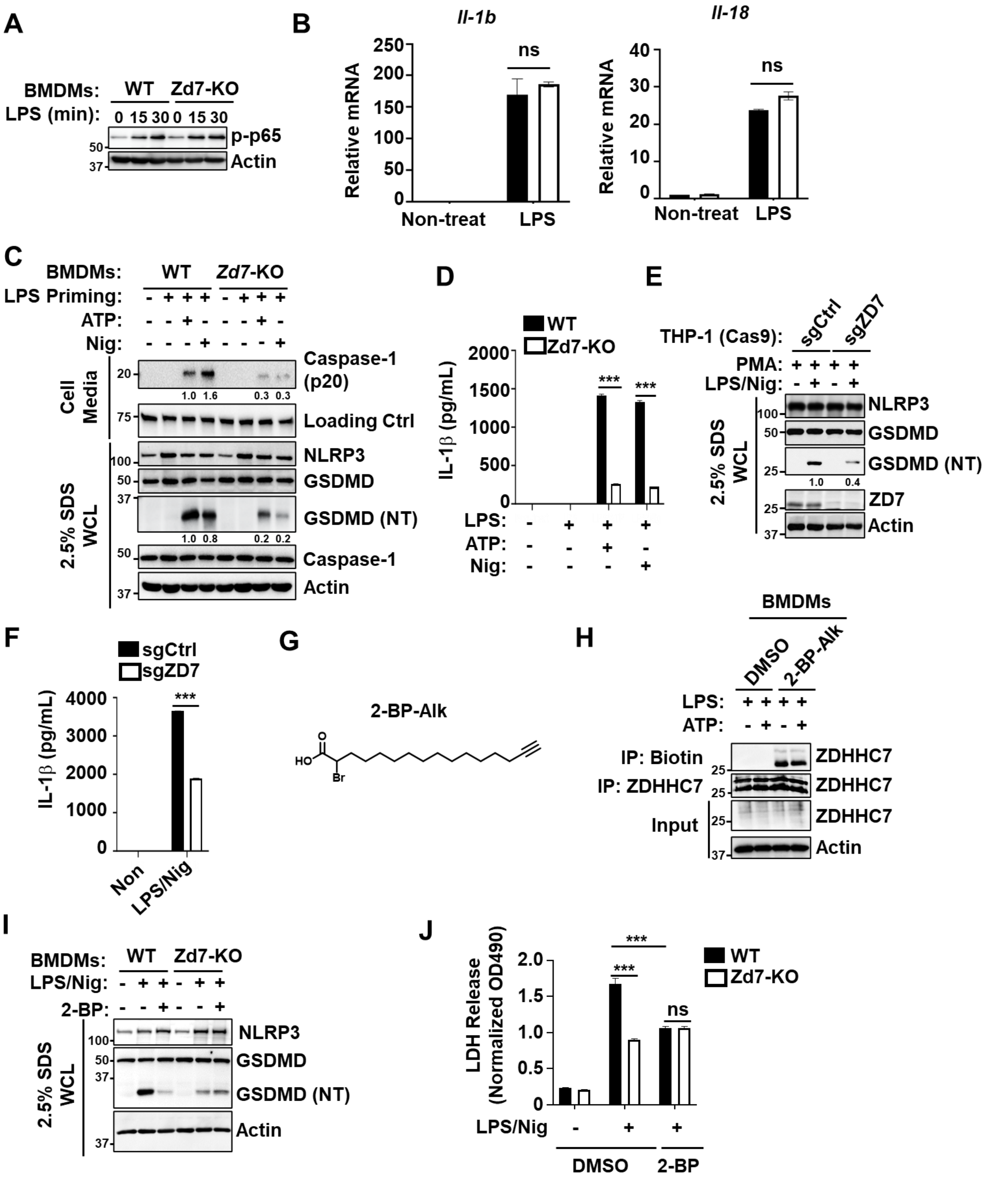

Figure S3. ZDHHC7-catalyzed *S*-palmitoylation is important for NLRP3 inflammasome activation, related to Figure 1.

(A) Phosphorylation of NF-κB (p65) determined by immunoblotting analysis with anti-phospho-p65 (Ser536) in wildtype (WT) and *Zdhhc7*-knockout (Zd7-KO) BMDMs that were treated with 100 ng/mL LPS for the indicated time. **(B)** Relative mRNA of *Il-1b* and *Il-18* determined by Q-PCR in wildtype (WT) and *Zdhhc7*-knockout (Zd7-KO) BMDMs that were treated with 100

ng/mL LPS for 6 h, mRNA was normalized to *β-actin*. **(C)** Immunoblot analysis of NLRP3, Caspase-1, GSDMD, and cleaved Caspase-1 (p20), cleaved GSDMD N-terminal domain (NT) in cell culture medium (top) and whole cell lysate (WCL, bottom) of WT and *Zdhhc7*-KO (Zd7-KO) BMDMs that were primed by LPS and activated with ATP or nigericin (Nig) as indicated. Cells were lysed with lysis buffer containing 2.5% SDS to get total proteins. **(D)** ELISA assay of IL-1β in cell culture media of WT and *Zdhhc7*-KO BMDMs in **(C)**. **(E)** Immunoblot analysis of NLRP3, GSDMD, and cleaved GSDMD N-terminal domain (NT) in *ZDHHC7*-deleted (sgZD7) and control (sgCtrl) THP-1 cells that were transduced with Cas9 nuclease. Cells were differentiated by PMA, primed with LPS, and activated with nigericin. Lysis buffer containing 2.5% SDS was used to get total proteins. **(F)** IL-1β determined by ELISA in cell culture media of *ZDHHC7*-deleted (sgZD7) and control (sgCtrl) THP-1 cells in **(E)**. **(G)** Chemical structure of 2-BP-Alk probe. **(H)** Immunoblot analysis showing 2-BP-Alk covalently bound to ZDHHC7 in LPS-primed and ATP activated BMDMs. 2-BP-Alk was incubated with BMDMs for 6 h, during which LPS was added for 4 h and ATP was added for 30 mins before cell collection. Whole cell lysate was conjugated with biotin-azide and then immunoprecipitated with streptavidin resin, or with anti-ZDHHC7 as control, ZDHHC7 was assessed by immunoblot. **(I)** Immunoblot analysis of NLRP3, GSDMD, cleaved GSDMD (NT) and Actin in wildtype (WT) and *Zdhhc7*-KO (Zd7-KO) BMDMs that were primed with LPS and activated with nigericin (Nig), with DMSO or 10 μM 2-BP treatment as indicated. **(J)** LDH release assay of BMDMs in cell culture medium showing cell pyroptotic level in (**I**). Data with error bars represent mean ± SEM. $*p < 0.05$, $**p < 0.01$, $***p < 0.001$ as determined by unpaired Student's t test.

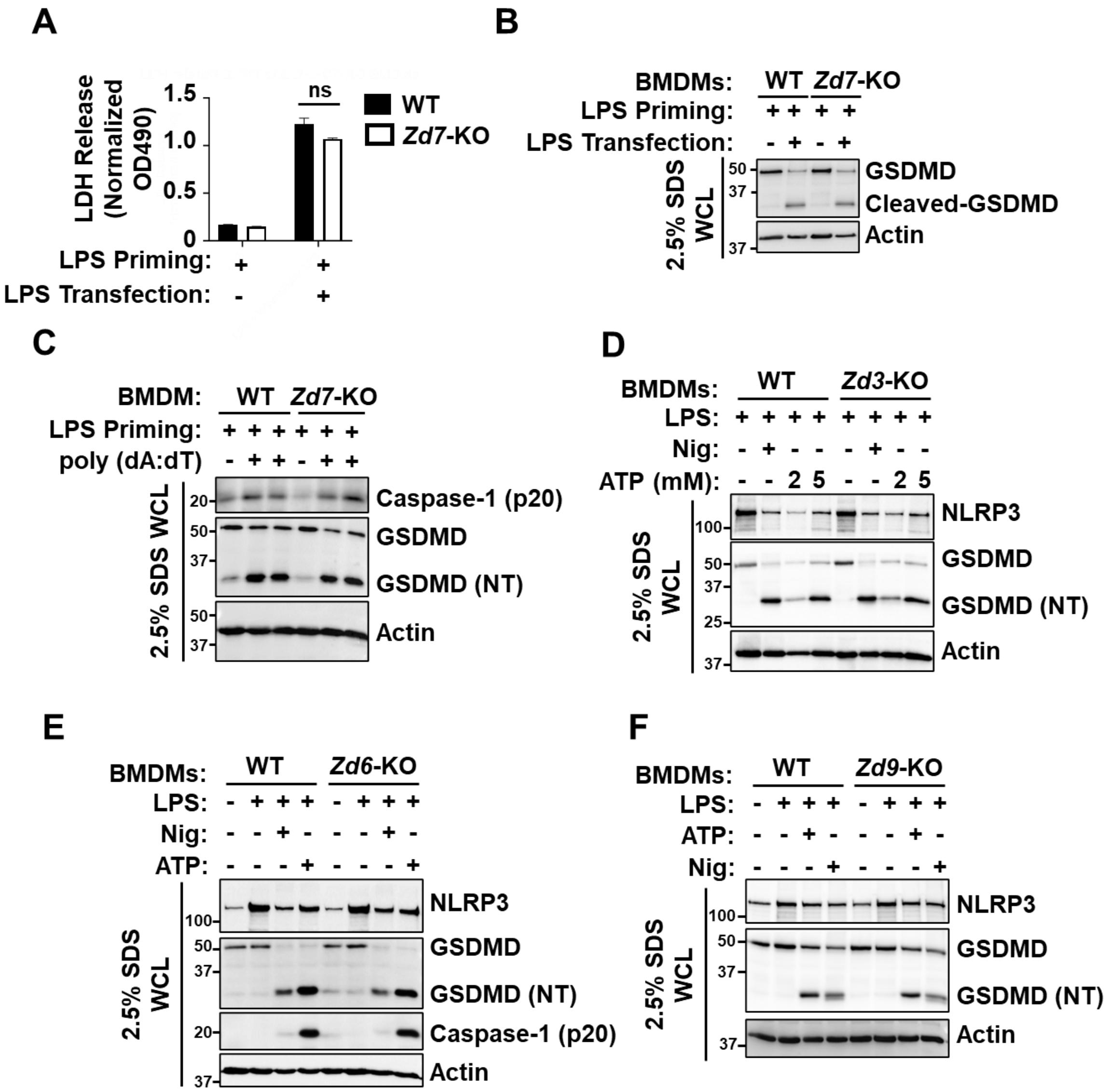

Figure S4. Zdhhc7 knockout in macrophage does not regulate non-canonical or AIM2 inflammasome activation, related to Figure 1.

(A) LDH release assay (pyroptosis) of non-canonical inflammasome activation in wildtype and *Zdhhc7*-KO BMDMs that were primed with 200 ng/mL LPS and activated by LPS transient transfection (1 μg/mL, 6 hours). **(B)** Immunoblotting analysis of GSDMD and GSDMD cleavage (NT) in (**A**). **(C)** Immunoblotting analysis of GSDMD, cleaved Caspase-1 (p20) and cleaved GSDMD (NT) for AIM2 inflammasome activation in wildtype and *Zdhhc7*-KO BMDMs that were primed with 200 ng/mL LPS and activated by poly(dA:dT) transfection (2 μg/mL, 6 hours). **(D-F)** Immunoblotting analysis of NLRP3, GSDMD and GSDMD cleavage (NT) in whole cell lysate (WCL) of wildtype (WT), Zdhhc*3*-KO (Zd3-KO, **D**), *Zdhhc6*-KO (Zd6-KO, **E**), or *Zdhhc9*-KO (Zd9-KO, **F**) BMDMs that were primed by LPS and activated with nigericin (Nig) or ATP as indicated. Cells and culture medium were lysed with lysis buffer containing 2.5% SDS to get total proteins. Data with error bars represent mean ± SEM. ∗p < 0.05, ∗∗p < 0.01, ∗∗∗p < 0.001 as determined by unpaired Student's t test.

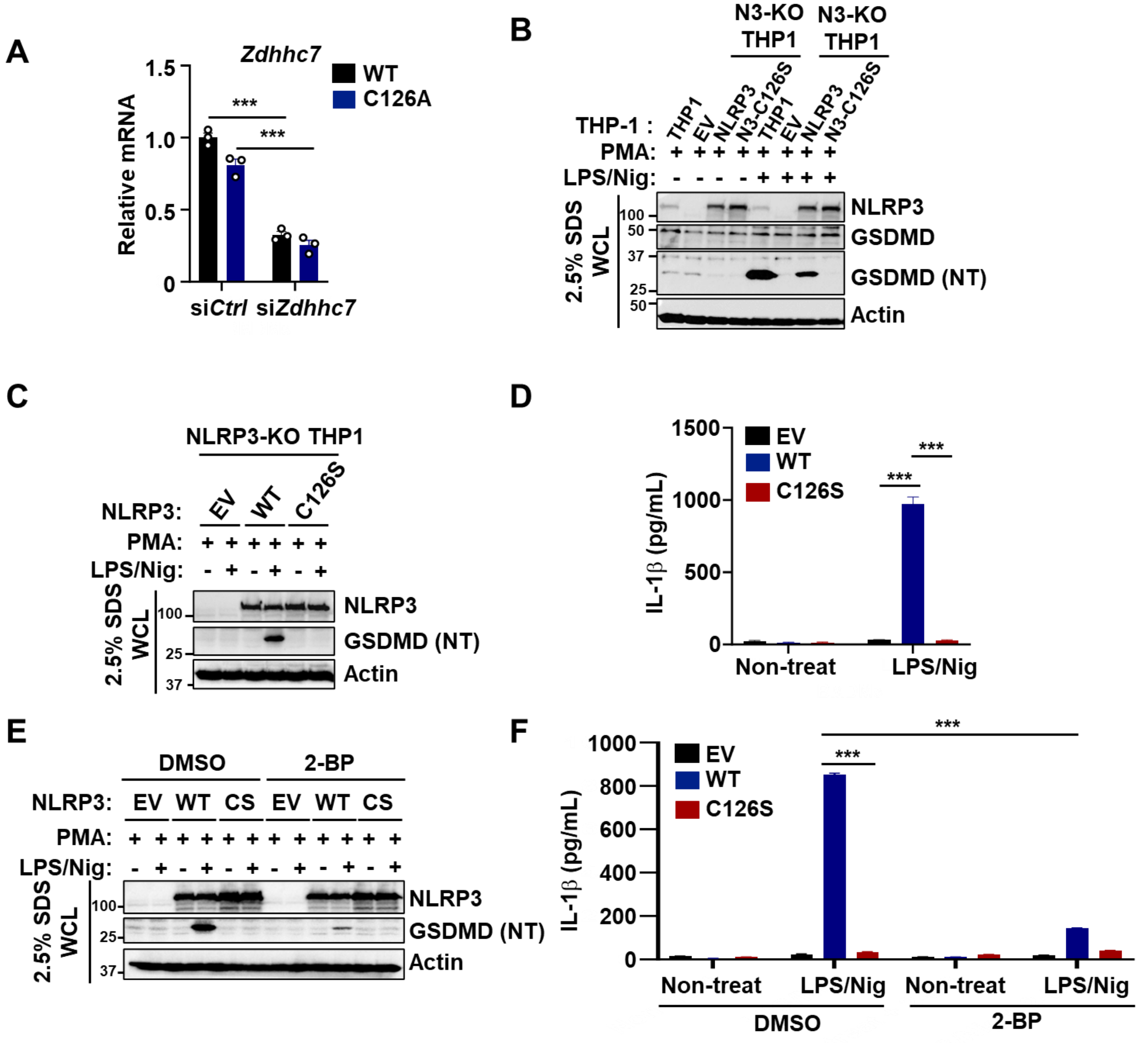

Figure S5. NLRP3 Cys126 mutant inhibited NLRP3 inflammasome activation in macrophages, related to Figure 2-3.

(A) Relative mRNA of *Zdhhc7* determined by Q-PCR in wildtype (WT) and *Nlrp3*-C126A (C126A) BMDMs that were knocked down with either control or *Zdhhc7* siRNA (si*Zd7*) as indicated. mRNA level was normalized to *β-actin*. **(B)** Immunoblot analysis of NLRP3, GSDMD, and cleaved GSDMD (NT) in PMA-differentiated wildtype or the NLRP3-KO (N3-KO) THP-1 cells that were reconstituted with wildtype or C126S mutant NLRP3 and stimulated with LPS and nigericin as indicated. **(C)** Replication of immunoblot analysis in (**B**). **(D)** ELISA determination of human IL-1β in the cell culture media in (**C**). **(E)** Immunoblot analysis of NLRP3 and GSDMD cleavage (NT) in PMA-differentiated NLRP3-KO THP-1 cells that were reconstituted with wildtype or C126S mutant NLRP3. Cells were PMA-differentiated, LPS primed, treated with DMSO or 10 μM 2-BP, and stimulated with 10 μM nigericin for 1 hour. **(F)** ELISA determination of human IL-1β in (**E**). Data with error bars represent mean ± SEM. $*p < 0.05$, $**p < 0.01$, $***p < 0.001$ as determined by unpaired Student's t test.

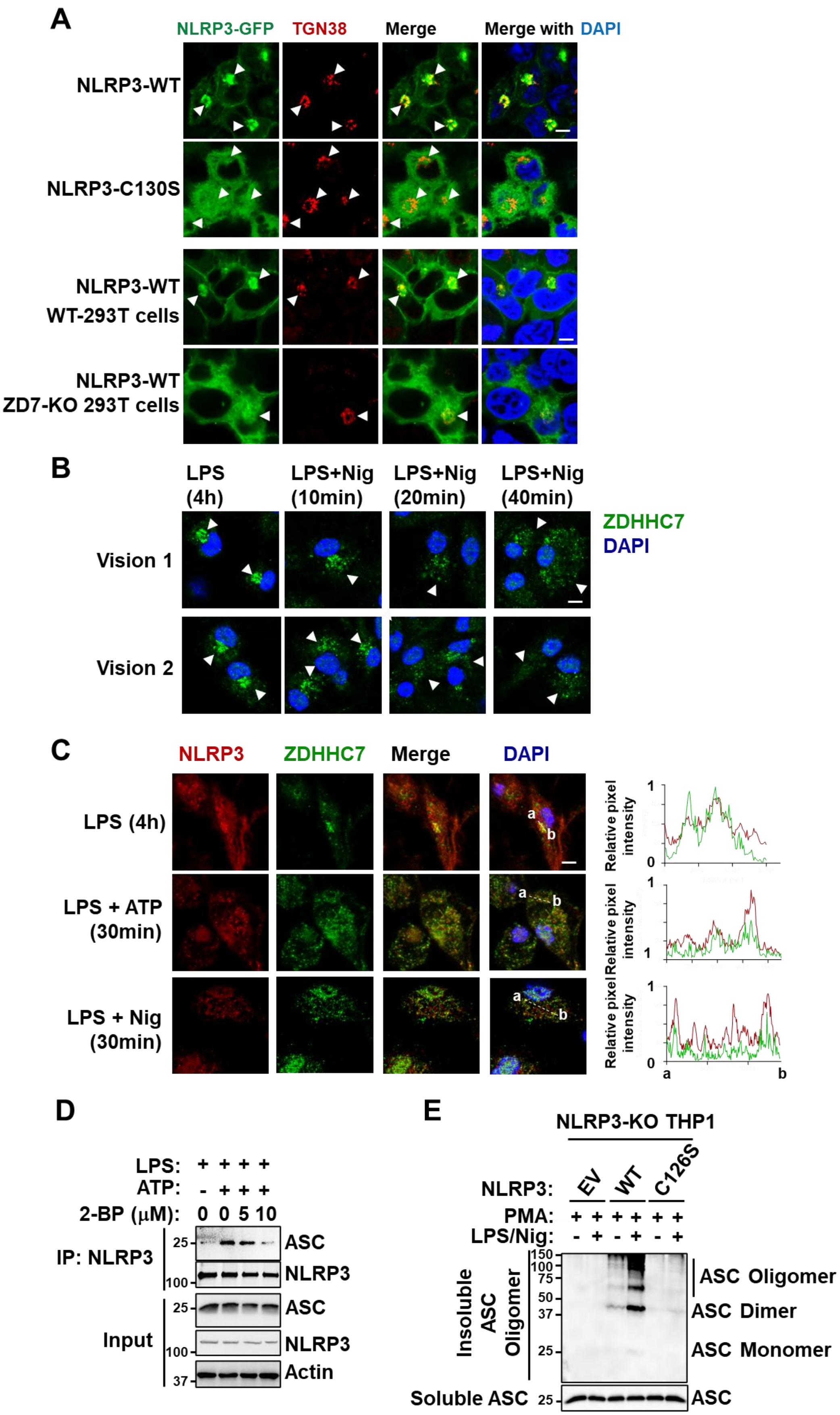

A
NLRP3-GFP
TGN38
Merge
Merge with DAPI
NLRP3-WT
NLRP3-C130S
NLRP3-WT
WT-293T cells
NLRP3-WT
ZD7-KO 293T cells
B
LPS (4h)
LPS+Nig (10min)
LPS+Nig (20min)
LPS+Nig (40min)
ZDHHC7
DAPI
Vision 1
Vision 2
C
NLRP3
ZDHHC7
Merge
DAPI
LPS (4h)
LPS + ATP (30min)
LPS + Nig (30min)
Relative pixel intensity
a
b
D
LPS:
ATP:
2-BP (μM): 0 0 5 10
IP: NLRP3
Input
ASC
NLRP3
Actin
E
NLRP3-KO THP1
NLRP3: EV WT C126S
PMA:
LPS/Nig:
Insoluble ASC Oligomer
ASC Oligomer
ASC Dimer
ASC Monomer
Soluble ASC
ASC

Figure S6. NLRP3 S-palmitoylation promotes NLRP3 locating on *trans*-Golgi network and ASC assembly, related to Figure 4-5.

(A) Representative confocal microscopic images of human wildtype and C130S mutant of human NLRP3 (NLRP3-GFP) in HEK 293T cells, or in *ZDHHC7*-KO (ZD7-KO) HEK 293T cells. WT NLRP3, but not the C130S mutant, colocalized with TGN marker TGN38. WT NLRP3 did not colocalize with TGN marker in *ZDHHC7*-KO cells. NLRP3 localization was shown as green, TGN38 antibody was used to stain *trans*-Golgi network, DAPI was used to stain the nucleus. Scale bar: 5 μm. **(B)** Representative confocal microscopic images of endogenous ZDHHC7 in BMDMs that were primed with 200 ng/ml LPS for 4 hours and activated with 10 μM nigericin as indicated. ZDHHC7 antibody was used to stain ZDHHC7 (green), DAPI was used to stain the nucleus. Scale bar: 5 μm. The images suggested Golgi-localized ZDHHC7 was dispersed during inflammasome activation in BMDMs. **(C)** Representative images showing the locations of endogenous NLRP3 and ZDHHC7 along with the DAPI signal in BMDMs that were primed with 200 ng/ml LPS for 4 hours and activated with 5 mM ATP or 10 μM nigericin (Nig) for 30 min as indicated. Representative curves (right) describe the distribution of relative fluorescence intensities for NLRP3 (red) and ZDHHC7 (green). Scale bar: 5 μm. Data represents at least two independent experiments. **(D)** Immunoblot analysis of NLRP3 interaction with ASC in mouse macrophage cell line J774A.1 treated with DMSO or 2-BP. Cells were primed with 200 ng/mL LPS for 4 h, then incubated with DMSO or 2-BP (5 and 10 μM) and activated with ATP (5 mM) for 30 min. **(E)** Immunoblot analysis of ASC oligomerization from insoluble protein fraction by DSS cross-linking in PMA-differentiated and LPS-primed THP-1 cells that were reconstituted with wildtype or C126S mutant of NLRP3 and activated with 10 μM nigericin for 1 h.

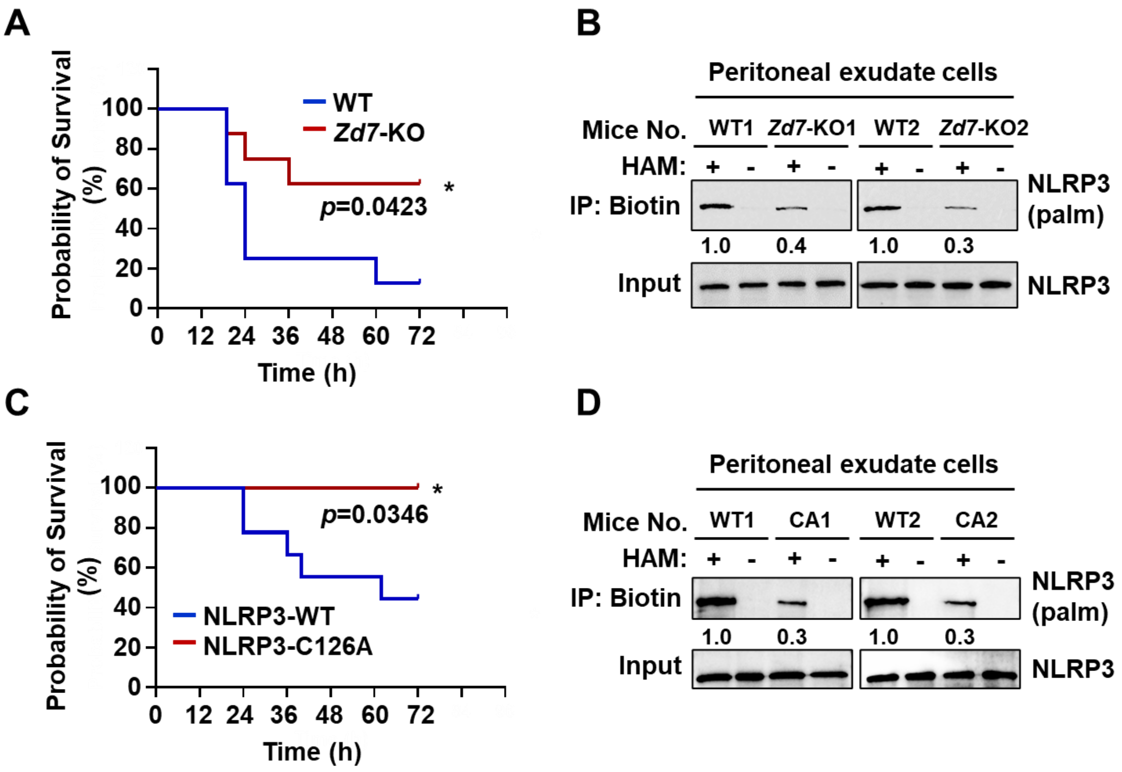

Figure S7. NLRP3 was *S*-palmitoylated on Cys126 by ZDHHC7 *in vivo*, related to Figure 6.

(A) Survival data of ZDHHC7 WT and KO mice in response to LPS challenge. The survival curve was statistically analyzed with Log-rank (mantel-cox) test (n = 8 for each gourp). **(B)** NLRP3 palmitoylation detection in peritoneal exudate cells (PEC) from ZDHHC7 WT and KO (*Zd7*-KO) mice with LPS-induced endotoxic shock by acyl-biotin exchange (ABE) assay. NLRP3 palmitoylation level was quantified and normalized to NLRP3 protein levels in the input samples. **(C)** Survival data of NLRP3 WT and C126A mice in response to LPS challenge. The survival curve was statistically analyzed with Log-rank (mantel-cox) test (n = 9 for WT and n = 6 for C126A group). **(D)** NLRP3 palmitoylation level in PEC from NLRP3 WT and C126A (CA) mouse with endotoxic shock was determined by ABE assay. NLRP3 palmitoylation level was quantified and normalized to NLRP3 protein levels in the input samples.

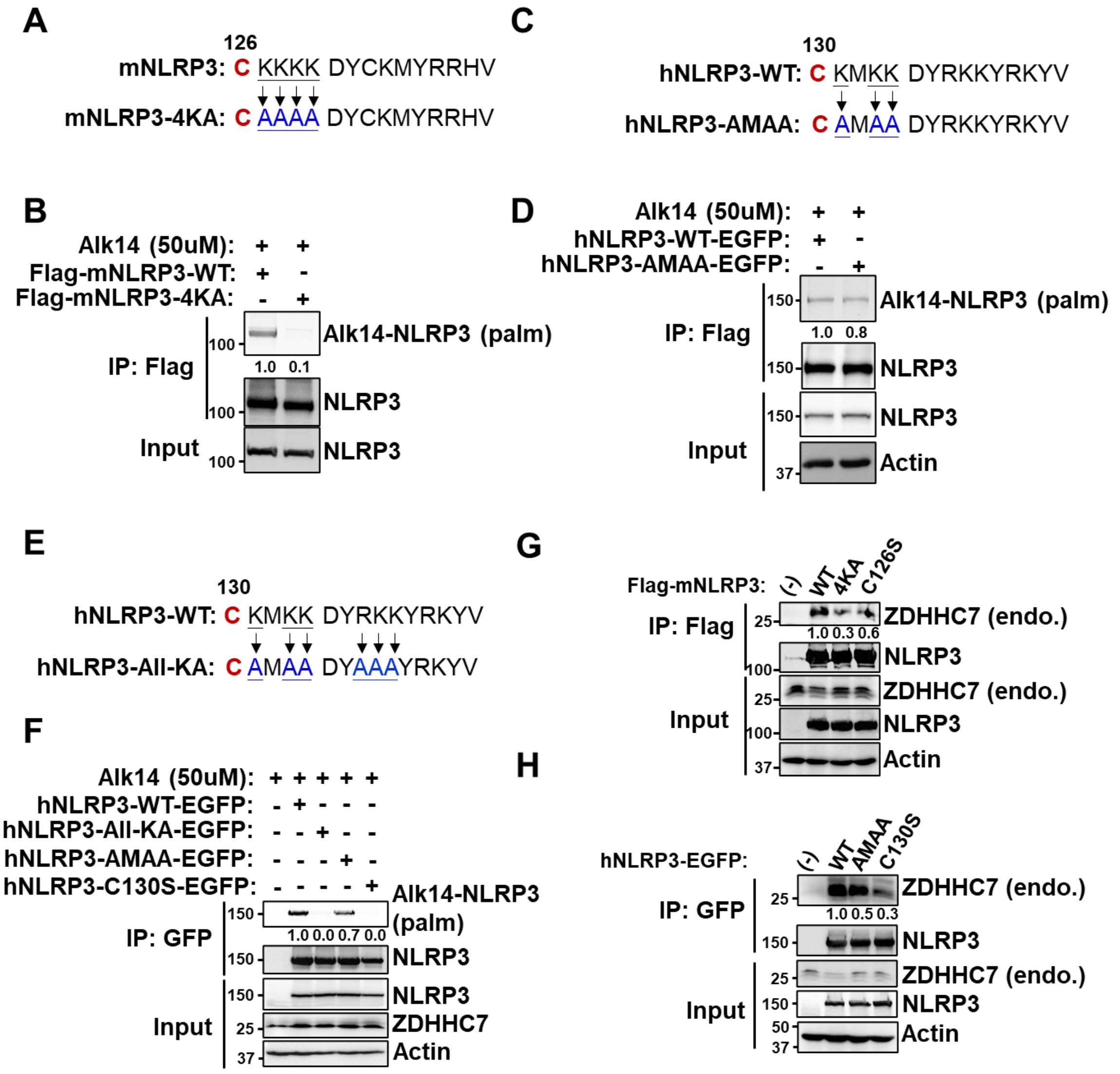

Figure S8. NLRP3 polybasic region is important for ZDHHC7-catalyzed NLRP3 Cy126 palmitoylation, related to Figure 4 and Figure 7.

(A) Schematic diagram of amino acids sequence for mouse NLRP3 protein polybasic region with lysine mutated to alanine (mNLRP3-4KA), which disrupts NLRP3 TGN localization. **(B)** Palmitoylation of wildtype (WT) or polybasic region demolition mutants (4KA) NLRP3 expressing in HEK 293T cells by Alk14 labeling and click chemistry assay. NLRP3 palmitoylation level was quantified and normalized to NLRP3 protein level. **(C)** Schematic diagram of amino acids sequence for human NLRP3 protein polybasic region with partially lysine mutated to alanine (hNLRP3-AMAA), which is not sufficient to prevent NLRP3 TGN localization. **(D)** Palmitoylation of human WT or AMAA mutant of NLRP3 in HEK 293T cells detected by Alk14 labeling and click chemistry assay. NLRP3 palmitoylation level was quantified as above. **(E)** Schematic diagram of amino acids sequence for human NLRP3 protein polybasic region with all nearby K/R mutated to alanine (hNLRP3-All-KA), which disrupts NLRP3 TGN localization. **(F)** Palmitoylation of human WT, All-KA, AMAA, or C130S NLRP3 in HEK 293T cells detected by

Alk14 labeling and click chemistry assay. NLRP3 palmitoylation level was quantified as above. **(G-H)** Immunoblotting assay of endogenous (endo.) ZDHHC7 interaction with mouse WT or 4KA NLRP3 (**G**), or human WT or AMAA NLRP3 (**H**) in HEK 293T cells. Cell lysate was immunoprecipitated with anti-Flag resin to pull-down NLRP3, endogenous ZDHHC7 was detected by immunoblot with anti-ZDHHC7 in the immunoprecipitation (IP) samples. The Co-IP ratio of ZDHHC7/NLRP3 was calculated and normalized to protein levels in input samples.

Supplementary Table 1. Sequences of Q-PCR Primers, related to Figure 1, S1, S3, and S5.

**Genes**	**Primers**	**Sequences (5' to 3')**
Mouse *Il-1b*	Forward	AAGCCTCGTGCTGTCGGACC
	Reverse	TGAGGCCCAAGGCCACAGGT
Mouse *Il-18*	Forward	GACAGCCTGTGTTCGAGGATATG
	Reverse	TGTTCTTACAGGAGAGGGTAGAC
Mouse *β-actin*	Forward	CGTGAAAAGATGACCCAGATCA
	Reverse	CACAGCCTGGATGGCTACGT
Mouse *Zdhhc1*	Forward	GCGCATGTCATTGAAGACCTGC
	Reverse	CACGCAGTTGTTGAGCCACTTG
Mouse *Zdhhc2*	Forward	AGTTCTGAGGCGAGCAGCCAAA
	Reverse	CAGACGGAACAATGATGACAGCG
Mouse *Zdhhc3*	Forward	TGGTGGGATTCCACTTCCTGCA
	Reverse	GCCTCAAAGCACAGCAGGATGA
Mouse *Zdhhc4*	Forward	CCGGTTTGGGCCGGTTC
	Reverse	CAGATAGCAGCTCCGCTTGG
Mouse *Zdhhc5*	Forward	ACACACCTCAGCCTGGCTACTA
	Reverse	ATGGCGGCTGATGTGCTACTGC
Mouse *Zdhhc6*	Forward	AGTCTGCCAAGCATACAAGGCG
	Reverse	CAACAGGAGGAAGAGCGTGAAC
Mouse *Zdhhc7*	Forward	GCTCTGTCTTCGGTTCATGCTC
	Reverse	CTCAAGGCACAGGAAGACCAAC
Mouse *Zdhhc8*	Forward	GTGCCCTATCAGTACAGAGGAC
	Reverse	GGTGCTGTCATCTGCCAGAGTA
Mouse *Zdhhc9*	Forward	CTGCTGTGAAGTGCTTTGTGGC
	Reverse	TCTGTGGCAACAGGCTACTGCT
Mouse *Zdhhc11*	Forward	CATCCAGCAGAGGAGAAAGAGC
	Reverse	TCGGCGAAAGAGTAGACACTGG
Mouse *Zdhhc12*	Forward	GTGCTGACCTGGGGAATCAC
	Reverse	CTGCACATTCACGTAGCCA

Mouse *Zdhhc13*	Forward	TGGTTCTAGCCTGGACATCCGA
	Reverse	CCATCGCCAAAGCCGAAACTGT
Mouse *Zdhhc14*	Forward	ACAGAAGAGGCTATGTCCAGCC
	Reverse	GCTCTGAATGCACTGGTCTTGG
Mouse *Zdhhc15*	Forward	AGAGACCTGAGGTCCAGAAGCA
	Reverse	AGACAGAACAGTGATGGCAGCG
Mouse *Zdhhc16*	Forward	TGATGCTGCCTTTGAGCCTGTC
	Reverse	ACCGAGTAGGTTCGGAGGATGA
Mouse *Zdhhc17*	Forward	CTTCCTTGCCAACAGCGTTGCT
	Reverse	TGAGGTCCAGACTTCCAGTCTC
Mouse *Zdhhc18*	Forward	GGAGACGGAACTACCGCTTCTT
	Reverse	GCTGGTGTCTTTTTCAGAGCGG
Mouse *Zdhhc19*	Forward	CGAGCGTGTTTGCTGCCTTCAA
	Reverse	AGGTGAGGATGAAGAGTGGTCC
Mouse *Zdhhc20*	Forward	GCAAACCAGAGTGACTACGTCAG
	Reverse	CAGCTCCATTCTCTAGCCACTG
Mouse *Zdhhc21*	Forward	CTGAGCTGCTTACTTGCTACGC
	Reverse	TGCCCATGAAGGCAGCTAGTCT
Mouse *Zdhhc22*	Forward	GCCTACATCTCCGCTGTCCTTT
	Reverse	ATGGCGAACCAGAGGTAGAGCA
Mouse *Zdhhc23*	Forward	GGATATGCGGTATCTGTGTACGG
	Reverse	GGTCAGCGATATTCCGTAAACCG
Mouse *Zdhhc24*	Forward	TCTACACAGTGGCTCTCCTGCT
	Reverse	AAAAGCAGCCCAGCACCACACA
Mouse *Zdhhc25*	Forward	CGTCACACCTACGGACTATGCT
	Reverse	AGTGGCAAGCTGTCCTCGGTAT
Human *ZDHHC7*	Forward	CTGACCGGGTCTGGTTCATC
	Reverse	CATGACGAAAGTCACCACGAA
Human *β-ACTIN*	Forward	GCAAGCAGGACTATGACGAG
	Reverse	CAAATAAAGCCATGCCAATC

Supplementary Table 2. Sequences of sgRNA for THP-1 stable cell line construction, related to Figure 1 and Figure 7.

**Target**	**Primers**	**Sequences (5' to 3')**
Human *ZDHHC7*	sgRNA#1	TCATGCAGCCATCAGGACAC
	sgRNA#2	CCGGTCAGCCACGTCAGCCT
	sgRNA#3	AGTCACCACGAAGTCTGCAT
Human *ZDHHC12*	sgRNA#1	CCTGGGGTCCTGGTGCGGAC
	sgRNA#2	GCTGACCTGGGGAATCACGC
	sgRNA#3	GTGCTCTTCCTGCACGATAC

REFERENCE

[S1]. Schmiedel, B.J., Singh, D., Madrigal, A., Valdovino-Gonzalez, A.G., White, B.M., Zapardiel-Gonzalo, J., Ha, B., Altay, G., Greenbaum, J.A., McVicker, G., et al. (2018). Impact of Genetic Polymorphisms on Human Immune Cell Gene Expression. Cell *175*, 1701-1715.e16. 10.1016/j.cell.2018.10.022.
